# Decreasing hospital burden of COVID-19 during the first wave in Regione Lombardia: an emergency measures context

**DOI:** 10.1186/s12889-021-11669-w

**Published:** 2021-09-03

**Authors:** Grosso Francesca Maria, Presanis Anne Margaret, Kunzmann Kevin, Jackson Chris, Corbella Alice, Grasselli Giacomo, Andreassi Aida, Bodina Annalisa, Gramegna Maria, Castaldi Silvana, Cereda Danilo, De Angelis Daniela, Covid-19 Lombardy Working Group, Castrofino Ambra, Del Castillo Gabriele, Crottogini Lucia, Tirani Marcello, Zanella Alberto, Salmoiraghi Marco

**Affiliations:** 1 Postgraduate School of Public Health, Department of Biomedical Sciences for Health, Via Pascal 36, University of Milan, Milan, Italy; 2 MRC Biostatistics Unit, University of Cambridge, East Forvie Building, Robinson way, Cambridge, UK; 3 Department of Statistics, University of Warwick, Mathematical Sciences Building, Academic Loop Road, Warwick, UK; 4 Fondazione IRCCS Ca’ Granda Ospedale Maggiore Policlinico, University of Milan, Via Francesco Sforza 28, Milan, Italy; 5 Welfare General Directorate, Regione Lombardia, Piazza Città di Lombardia 1, Milan, Italy

## Abstract

**Background:**

The aim of this study is to quantify the hospital burden of COVID-19 during the first wave and how it changed over calendar time; to interpret the results in light of the emergency measures introduced to manage the strain on secondary healthcare.

**Methods:**

This is a cohort study of hospitalised confirmed cases of COVID-19 admitted from February–June 2020 and followed up till 17th July 2020, analysed using a mixture multi-state model. All hospital patients with confirmed COVID-19 disease in Regione Lombardia were involved, admitted from February–June 2020, with non-missing hospital of admission and non-missing admission date.

**Results:**

The cohort consists of 40,550 patients hospitalised during the first wave. These patients had a median age of 69 (interquartile range 56–80) and were more likely to be men (60%) than women (40%). The hospital-fatality risk, averaged over all pathways through hospital, was 27.5% (95% CI 27.1–28.0%); and steadily decreased from 34.6% (32.5–36.6%) in February to 7.6% (6.3–10.6%) in June. Among surviving patients, median length of stay in hospital was 11.8 (11.6–12.3) days, compared to 8.1 (7.8–8.5) days in non-survivors. Averaged over final outcomes, median length of stay in hospital decreased from 21.4 (20.5–22.8) days in February to 5.2 (4.7–5.8) days in June.

**Conclusions:**

The hospital burden, in terms of both risks of poor outcomes and lengths of stay in hospital, has been demonstrated to have decreased over the months of the first wave, perhaps reflecting improved treatment and management of COVID-19 cases, as well as reduced burden as the first wave waned. The quantified burden allows for planning of hospital beds needed for current and future waves of SARS-CoV-2 i.

**Supplementary Information:**

The online version contains supplementary material available at 10.1186/s12889-021-11669-w.

## Introduction

On the 9th of January 2020, the health authorities in China reported that a novel strain of coronavirus, later named SARS-CoV-2, was the causative agent for many of the severe acute respiratory syndromes occurring in the area of Wuhan [1]. In response to the emerging situation, several pre-pandemic measures were implemented by the Italian Ministry of Health and by Regione Lombardia.

In Lombardia, seventeen “first-responder hub hospitals” were selected to be part of the Infectious Disease Hospital Network and charged with admission of suspected cases, because of their expertise in infectious diseases or their membership of the Venous-venous ECMO Respiratory Failure Network [2]. General Practitioners and Family Pediatricians received training on surveillance activities [3]; temperature scanners were set up in airports to screen passengers from in-bound flights; and an Operations Room for Emergencies was organised to manage emergency calls from suspected Covid-19 patients [4].

On 20/02/2020 the first Italian patient was diagnosed with Covid-19 in the Lombardia town of Codogno, which soon became the Italian epicenter of the pandemic. The number of confirmed infected patients rose to 403 by the following week, with 213 patients admitted to hospitals [5]. This abrupt rise in the number of confirmed cases, peaking eventually on the 20th of March 2020, put a large strain on the healthcare system in Lombardia [6], an Italian region of approximately 10 million inhabitants of whom 41% over 55 years of age (Additional file 1: Appendix A.1.1). The growing need for hospital capacity led to non-urgent procedures being cancelled (48 h after the first case) and to expanding the number of beds, especially in the ICU wards, where the pre-crisis capacity was around 720, immediately raised to 861 [2]. Regional Coordination Units were created (Additional file 1: Appendix A.1.3) to manage the crisis. The Regional Unit of Coordination for Hospital Admission collected data on the number of vacant beds daily and redirected patients with the purpose of redistributing the burden equally among the 17 first-responder hub hospitals (the “hub-and-spoke” model) especially involved during the initial phase of the pandemic and then among all hospitals in the region. A total of 482 ICU beds were made available over 18 days from the 20th February [2].

All of the enacted emergency measures likely impacted on the ability of the healthcare system of Lombardia to provide care. The aims of this study are: to estimate how mortality risk and progression of patients through hospital changed through the first wave; to interpret these changes in relation to the emergency measures implemented; and to inform management and relevant models of the next waves, currently in progress. The burden is quantified, using a multi-state modelling approach [7], in terms of risks of progression through hospitals of Covid-19 patients, together with lengths of stay in hospital and ICU.

## Methods

### Study participants

The Covid-19 Regional Database (Additional file 1: Appendix A.1.3) is an integrated database collecting data from laboratories, hospitals and Local Healthcare Agencies and comprises detailed, but pseudo-anonymised, retrospective individual-level data on the cohort of all individuals in Lombardia diagnosed with Covid-19 during the first wave from February to June 2020 [8–10].

Data, as extracted on 5th August 2020, include 95,777 records, representing 95,354 individuals with confirmed COVID-19 disease in 2020, observed from 1st December 2019 to 17th July 2020. The dataset records age, gender, Local Healthcare Agency district of Lombardia, co-morbidities, symptoms, whether the individual is a healthcare worker or care home resident, whether or not the individual was hospitalised, and details of the admitting hospital if the individual was hospitalised. For each individual, dates of symptom onset, positive laboratory test, hospital admission, ICU admission, ICU discharge, hospital discharge, recovery and death are recorded. Excluding duplicate records and records with inconsistent, missing or invalid dates leaves 94,474 individuals, of whom 46,609 were hospitalised. Finally, excluding the 12.4% of hospitalised patients with missing hospital of admission leaves 40,550 individuals in the dataset.

### Multi-state model

The progression of patients through hospital can be represented by a multi-state model with states Hospital (ward entered on admission date), ICU (entered at ICU admission), Post-ICU (entered after ICU discharge), Discharge (entered on date of hospital discharge), and Death (entered on date of death) (Additional file 1: Appendix Fig. A.2). The outcomes of interest are: the probabilities of entering each next state (a “next event”) given the current state; the probabilities of entering a final state (either a Death or Discharge event) given hospital admission (“*hospital-fatality risk*”, defined in Additional file 1: Appendix A.4) or given the current state; corresponding times to each next event, conditional on experiencing the next event, i.e. the lengths of stay in each state; and the total length of stay (LoS) in hospital.

The multi-state model is implemented as a “mixture model” [7, 11], combining multinomial or binomial logistic regression of probabilities of different pathways through hospital on covariates with parametric time-to-event analyses for the time taken to move from one state to the next (each LoS), conditional on the next event occurring. Analyses are carried out in R 3.6.3 using the *flexsurv* package [7].

In the Covid-19 Regional Database, outcomes/next events are missing for < 1% of individuals in the Hospital and ICU states, and for 15% of individuals in the Post-ICU state (Additional file 1: Appendix Fig. A.1). It is unknown why outcomes are missing, i.e. whether they had not happened by the end-date of the data, 17th July, (“right-censoring”), or whether they had not been recorded (“missing data/loss to follow-up”). Since it is impossible to distinguish between these possibilities, two alternative assumptions are made: (1) the missing outcomes are ignorable, i.e. individuals with missing outcomes have the same distribution of outcomes as those observed; (2) these individuals are right-censored at a time *t* after their last observed event. In this second case, the parametric time-to-event models account for the censoring, and typically *t* would be the length of time till the end of the dataset, i.e. till 17th July. However, we judge that it is implausible that all of those with missing outcomes are still in hospital by mid-July, so instead make the conservative assumption that *t* is 1 day. The results are very similar under the two assumptions (Additional file 1: Appendix A.3), so in what follows, only the results under assumption [2] are reported. As we consider discharge a final outcome, we ignore the 2.02% of discharged patients who are observed to die after discharge (Additional file 1: Appendix Fig. A.1).

We consider four models with different covariate combinations: (a) no covariates; (b) month of hospital admission; (c) hospital bed capacity; (d) both month of hospital admission and hospital bed capacity. Bed capacity is defined in terms of numbers of both hospital and ICU beds (Additional file 1: Appendix Table A.3). Point estimates and 95% confidence intervals (CIs) are reported for each probability of interest, as well as for the median and interquartile ranges (IQRs) of each time-to-event distribution. The confidence intervals represent parameter uncertainty, whereas the median and IQRs summarise the heterogeneity across individuals in the time-to-event distributions.

These models do not account for individual patient characteristics, which are explored in a companion paper [12].

All methods were performed in accordance with the Declaration of Helsinki.

## Results

### Dataset description

The cohort consists of 40,550 individuals who underwent hospitalization during the first wave. Their median age was 69 (interquartile range [56–80]); the proportion of men (60%) was higher than women (40%); most (69%) were admitted in March; 64% had at least one co-morbidity; 4% were care home residents; 4% were healthcare workers; and most (71%) were admitted to hospitals with a large bed capacity. Further covariate summaries for these patients are shown in Additional file 1: Appendix (Table A.4).

### Overall results

**Fig. 1:**
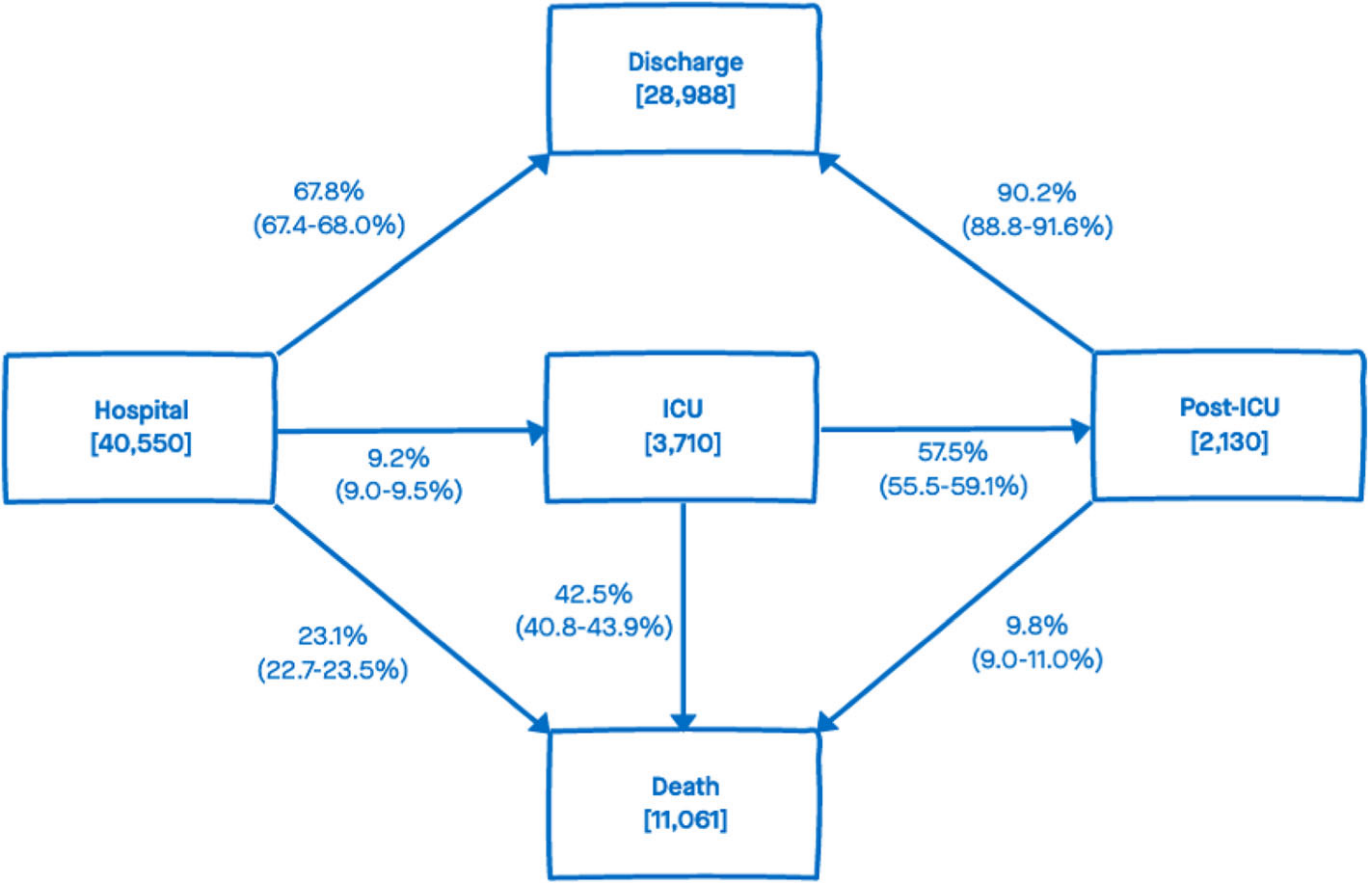
Multi-state model with estimated risks (point estimate and 95% CI in brackets) of moving from current states to next events. The numbers in each state in square brackets are the observed numbers of patients reaching each state. These observed numbers do not include the numbers of patients with missing next events (< 1% in the Hospital and ICU states, 15% in the Post-ICU state), nor the ignored 2% of patients who died after being discharged from hospital. In contrast, the estimated risks account for the missing next events, assuming they are censoring at 1 day after the patients’ last observed events

Figure 1 shows the specific hospital-fatality risks from each state: 23.1% (22.7–23.5%) from hospital without ICU; 42.5% (40.8–43.9%) from ICU; and 9.8% (9.0–11.0%) from post-ICU. When averaged over all pathways through hospital, the overall hospital-fatality risk was 27.5% (95% CI 27.1–28.0%).

The overall median (95% CI of median) LoS in hospital, averaged over all pathways through hospital and final outcomes was 10.4 (10.1–10.9) days. Among surviving patients, median LoS in hospital was 11.8 (11.6–12.3) days, while those whose final outcome was death had a median time to death of 8.1 (7.8–8.5) days.

The probability of ICU admission was 9.2% (9.0–9.5%) and the corresponding median time from hospital admission to ICU admission was 3.5 (3.3–3.6) days. The median LoS in ICU was 11.0 (10.7–11.6) days: 12.6 (12.2–13.2) days for survivors and 9.6 (9.3–10.1) days for non-survivors. The median LoS in a post-ICU ward was 18.0 (17.1–18.7) days: 18.8 (18.2–19.5) days for survivors and 10.1 (8.9–11.9) days for non-survivors. By pathway through hospital, median LoS in hospital was: 10.8 (10.3–10.9) days for surviving patients who were not admitted to ICU (hospital-discharge); 40.7 (40.1–41.8) days for surviving patients who were admitted ICU (hospital-ICU-postICU-discharge); 6.9 (6.6–7.1) days for non-survivors who were not admitted to ICU (hospital-death); 15.1 (14.8–15.9) days for patients who died in ICU (hospital-ICU-death); and 31.7 (30.0–33.0) days for patients who died in a post-ICU ward (ward-ICU-postICU-death).

### By month of admission

**Fig. 2:**
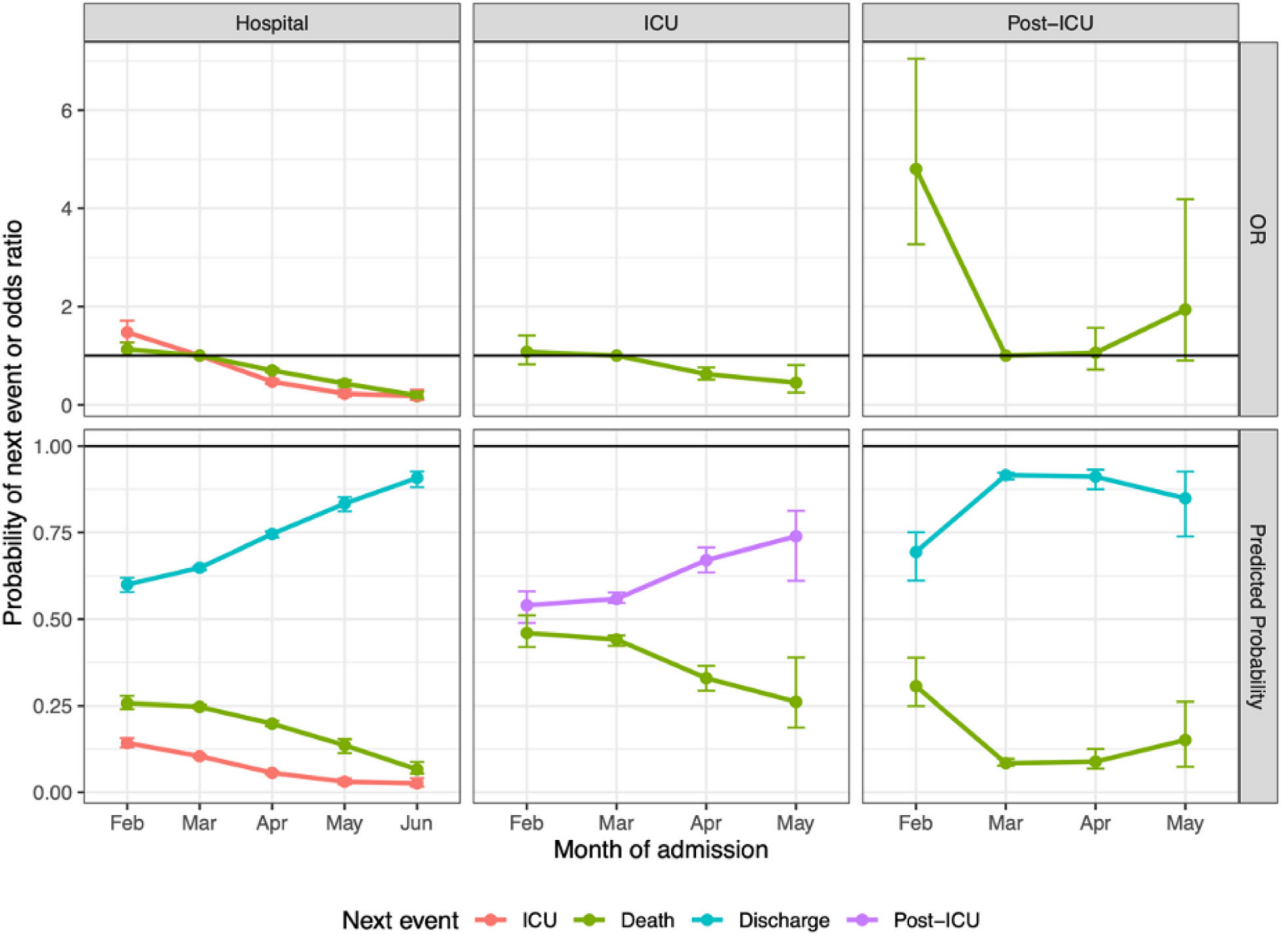
Estimated odds ratios (odds of next event in each month compared to the odds of next event in March) and predicted probabilities of moving from the current state (columns) to next events (colours). Points are point estimates and vertical bars represent 95% confidence intervals. Note that for the ICU and post-ICU states, the observed numbers of events in June were small, so the “May” month includes events from both May and June combined. Note also that odds ratios are presented for 2/3 or 1/2 of the next events, since the probability of the remaining event (discharge in all three columns) is just defined as 1 minus the other probabilities

Figure 2 shows the estimated odds ratios of risk of next events by month compared to a baseline of March, together with corresponding predicted probabilities of next events, demonstrating the overall decreasing trend in severe events. The probability of ICU admission from a hospital ward decreased from 14.3% (13.0–16.0%) in February to 2.6% (1.7–3.6%) in June; the hospital-fatality risk without ICU decreased from 25.7% (24.1–27.6%) in February to 6.6% (5.0–8.0%) in June; and the ICU-fatality risk decreased from 46.0% (41.5–52.8) in February to 26.1% (17.6–36.4%) in May–June.

The trend in fatality risk is more uncertain from the post-ICU state, with only February (at 30.6% [23.5–37.6%]) significantly higher than March (8.4% [7.6–9.7%]), and the post-ICU-fatality risk estimated to be 15.1% (7.8–29.9%) in May–June. Assuming the same risks of death from ICU or post-ICU in May and June, the overall hospital-fatality risk, regardless of path through hospital, is estimated to have steadily decreased from 34.6% (32.5–36.6%) in February to 29.8% (29.3–30.3%) in March, 22.0% (21.1–23.0%) in April, 14.7% (13.3–15.9%) in May and 7.6% (6.3–10.6%) in June.

The overall median LoS in hospital, regardless of outcome, decreased from 21.4 (20.5–22.8) days in February to 5.2 (4.7–5.8) days in June. Among survivors, median LoS in hospital, whether or not they had an ICU admission, decreased from 24.6 (22.8–26.1) days in February to 5.1 (4.8–5.8) days in June. Non-survivors had slightly shorter median LoS, decreasing from 17.8 (17.1–19.9) days in February to 4.7 (3.3–6.0) days in June.

**Fig. 3:**
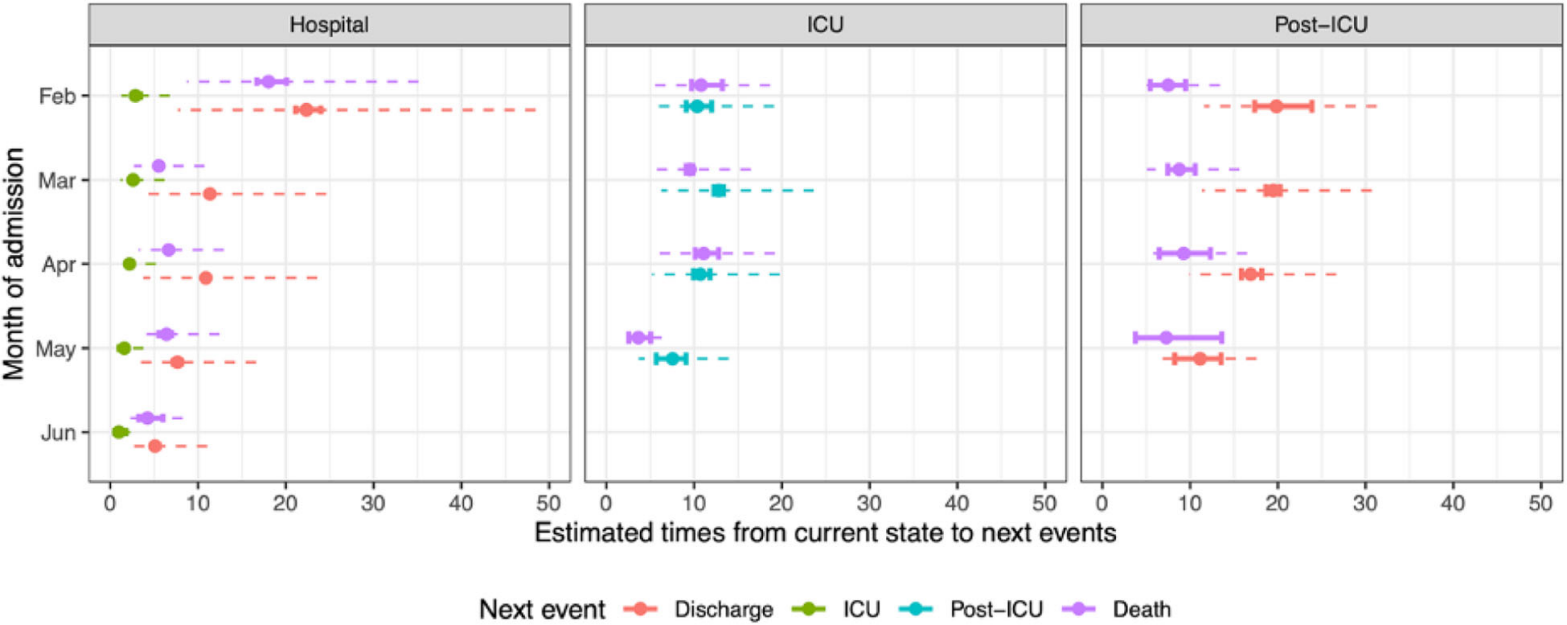
Summaries of the distributions of times from current state (columns) to next events (colours), by calendar month of admission. The 95% CI of the median times (solid lines) represent uncertainty in the estimate, whereas the inter-quartile range of the distribution (dashed lines) represents heterogeneity in the population

The median times spent by survivors in different stages of hospital (pre-ICU, ICU, and post-ICU) also appear to have decreased with calendar month of admission (Fig. 3). The median time to ICU admission decreases from 2.8 (2.5–3.3) days in February to 1.0 (0.6–1.6) days in June. Median LoS in hospital among survivors who were not admitted to ICU reduced from 22.4 (21.1–23.6) days in February to 5.1 (4.7–5.6) days in June; median LoS in ICU among survivors reduced from 10.3 (9.1–13.0) days in February to 7.5 (6.6–9.7) days in May–June, although it was longest in March, at 12.8 (12.3–13.4) days. Heterogeneity amongst survivors in their lengths of stay also appears to have decreased with month of admission (dashed lines in Fig. 3).

There is no clear trend in non-survivors who are not admitted to ICU: the median time to death is largest in February, at 18.0 (16.4–19.8) days, dropping to 5.5 (5.4–5.6) days in March, but then doesn’t appear to change significantly from March through to June. Similarly, the times to death from ICU and from a post-ICU stay do not appear to vary much with month of admission.

### Effect of hospital bed capacity

**Fig. 4:**
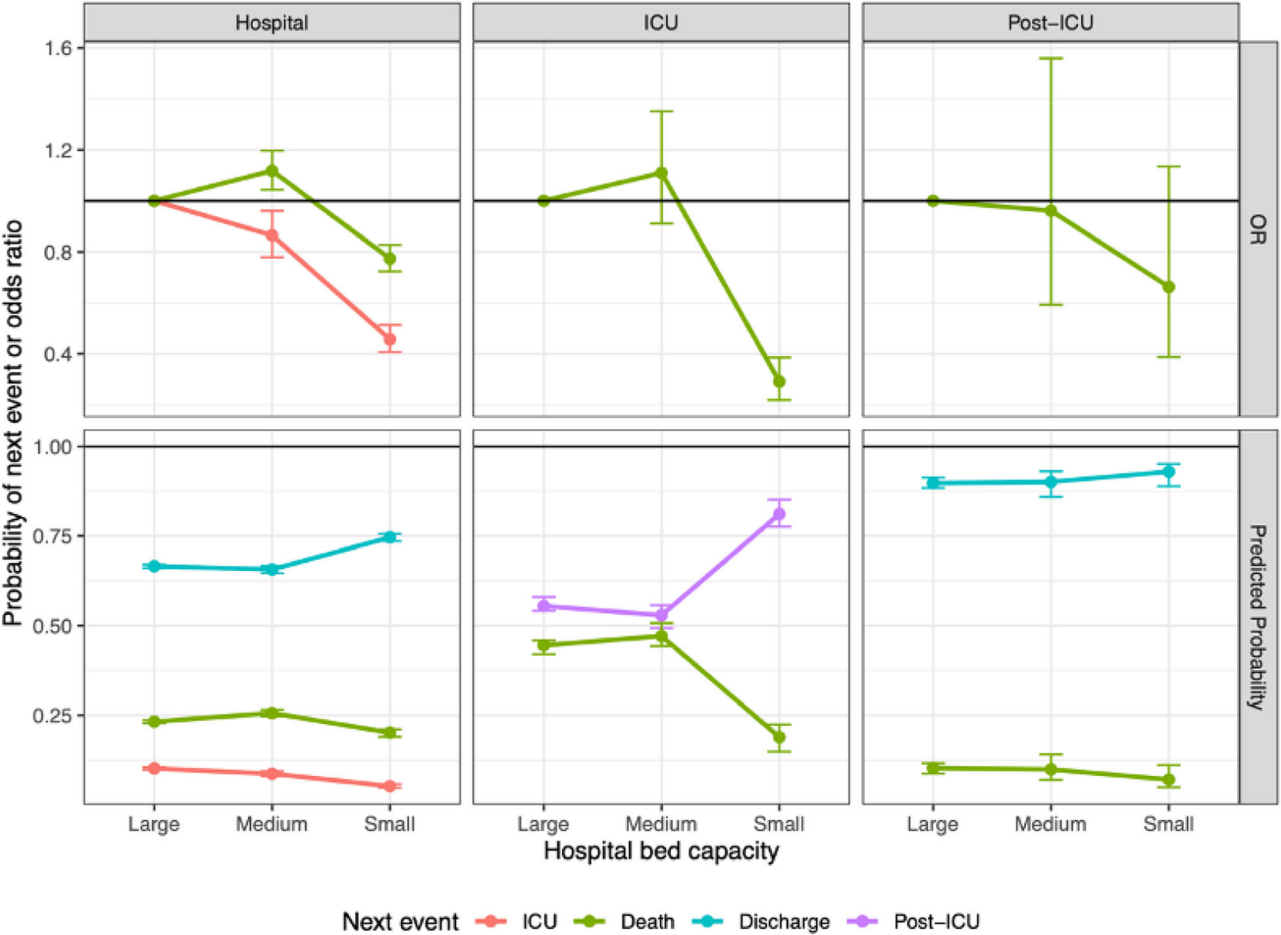
Estimated odds ratios (relative to large hospitals) and predicted probabilities of moving from the current state (columns) to next events (colours)

Hospital size, in terms of bed capacity, has a (unadjusted) significant effect on the probabilities of next events from hospital admission: the smallest hospitals have both the lowest risk of ICU admission (odds ratio 0.450 [0.400–0.506] relative to the largest) and a slightly lower risk of death without ICU admission (odds ratio 0.773 [0.723–0.827]) (Fig. 4). These lower risks correspond to a higher probability of discharge among small hospitals (Fig. 4). However, note that the majority of the smallest hospitals have zero ICU beds, so the lower risk of ICU admission may be an artefact of not having the capacity. The risk of death in ICU is also significantly smaller in the smallest hospitals compared to the largest (odds ratio 0.292 [0.220–0.386]), but may again be an artefact of the lower ICU capacity. Hospital bed capacity has no significant effect on risks of next events after a post-ICU stay. These risks combine to result in a hospital-fatality risk that is lowest in small hospitals: 21.4% (20.6–22.3%) compared to 30.2% (29.0–31.5%) and 28.4% (27.9–28.9%) in medium and large hospitals respectively.

**Fig. 5:**
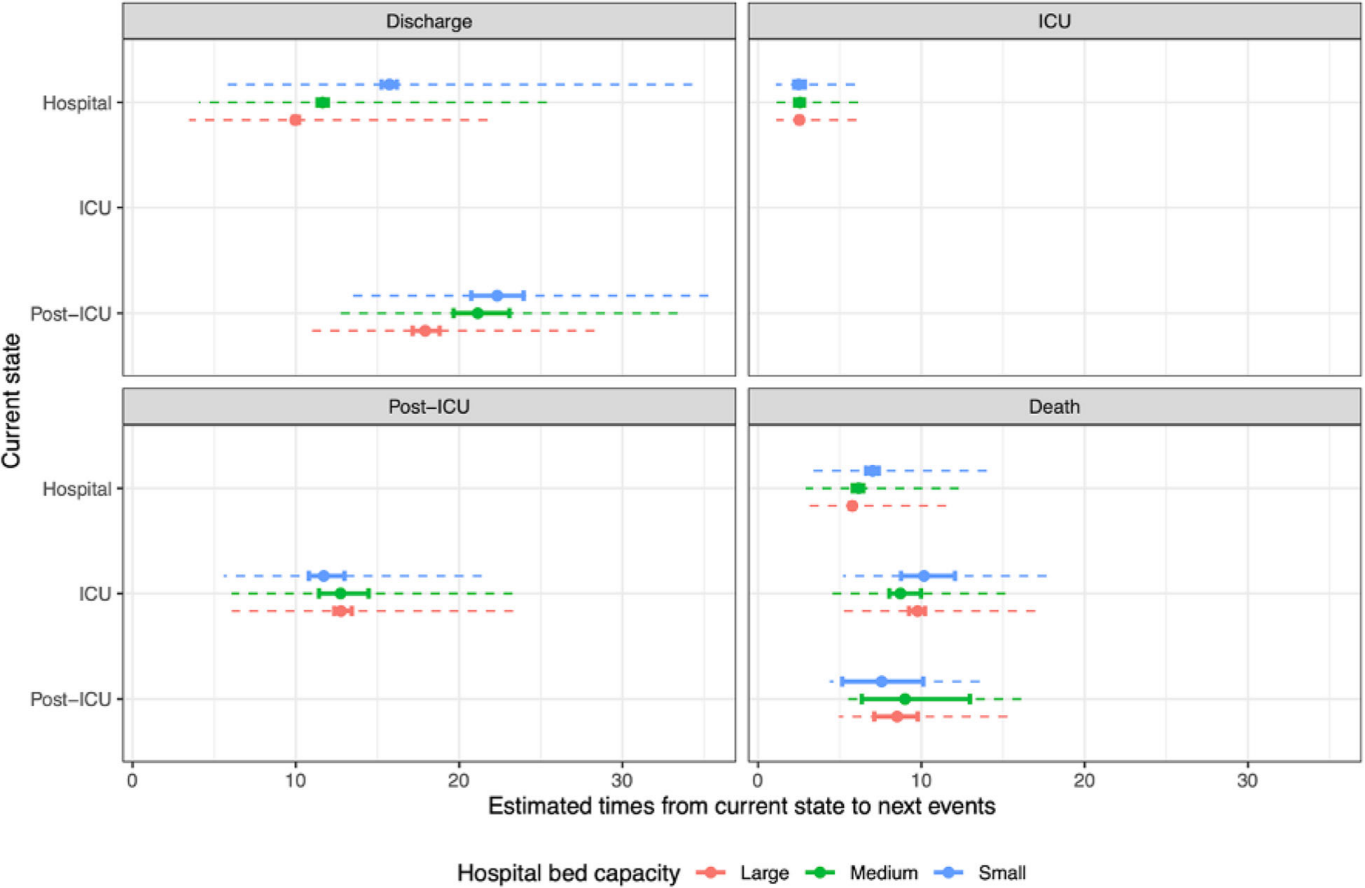
Summaries of distributions of lengths of stay in hospital, by current state (y-axis), next event (panels) and hospital bed capacity (colours). The 95% CI of the median times (solid lines) represent uncertainty in the estimate, whereas the inter-quartile range of the distribution (dashed lines) represents heterogeneity in the population

LoS for survivors in hospital without ICU and in a post-ICU ward become shorter as hospital bed capacity becomes larger, with small but significant effects (Fig. 5, top-left panel), whereas LoS in ICU does not vary significantly (Fig. 5, bottom-left panel). The effect of hospital bed capacity on time to death from hospital without ICU is marginally significant, but with only a small effect resulting in only a day’s difference between small and large hospitals. In ICU and post-ICU wards, the time to death does not vary by bed capacity (Fig. 5, bottom-right panel). Averaged over all pathways through hospital, median LoS is 13.7 (13.2–14.2) days in small hospitals, 10.4 (10.1–10.7) days in medium hospitals, and 9.7 (9.4–9.9) days in large hospitals. This effect of hospital bed capacity appears significant for survivors (11.1 [10.8–11.7], 13.0 (12.3–13.3) and 16.6 (16.0–17.8) days respectively for large, medium and small hospitals), but not for non-survivors: time to death is 7.0 (6.6–7.3), 7.5 (6.7–7.6) and 7.6 (6.8–8.0) days respectively.

When adjusting for both hospital bed capacity and month of admission simultaneously, our findings are similar to the univariable results in Sections 3.3 and 3.4 (Additional file 1: Appendix A.3.4).

## Discussion

This analysis has demonstrated substantial changes in the hospital burden of COVID-19 disease in Lombardia over the first wave. Quantifying burden is paramount for contingency planning in terms of beds, equipment and staff needed in hospitals. Figure 1 shows the proportion of patients that will most likely experience each outcome after admission, that is 9.2% (9.0–9.5%) will undergo ICU admission, a proportion that is much lower than the 24% reported by Boelle et al. [13]. In contrast, 67.8% (67.4–68.0%) will be discharged without ICU admission, a number that is comparable to the 63% found by Boelle et al. The outcome of death without ICU admission is also slightly higher (23.1% [22.7–23.5%]) when compared to Boelle et al. (13%).

At the peak of the first wave in March, the number of hospitalized patients rose to 7387, 37.15% of the reported positive cases (19,884, 10), although this ratio is affected by a selection bias resulting from the early policy of preferential testing of symptomatic cases. Preliminary work on extending the multi-state model to estimate hospital admission risk amongst cases with symptoms suggests 45.7% (45.2–46.1%) of symptomatic cases in Milano ATS required hospital admission in the first wave. Making the admittedly strong assumption that these results are applicable more widely to Lombardia, 45 beds might be expected to be needed for every 100 symptomatic cases. Moreover, our results suggest that the majority of beds (almost 70% of the total hospital admissions) should be planned for wards, while around 10% of hospital admissions are expected to require ICU beds; post-ICU beds should number at least 60% of ICU beds (Fig. 1).

Length of stay (LoS) in hospital is also an important measure to factor in when planning for an emergency. Our estimates of both overall median length of stay in hospital (10.4 days (10.1–10.9)) and in ICU (11.0 days (10.7–11.6)) are comparable with the LoS reported in the systematic review of Rees et al. [14] covering publications between 1 January 2020 and 12 April 2020. The authors found a hospital LoS of 4 days (1–9) outside China compared to 14 days (10–19) in China; and an ICU LoS of 7 days (4–11 days) outside China compared to 8 days (5–13) in China. The longer estimated LoS we found for surviving patients, when compared to patients whose final outcome was death, seems consistent with observations in the review but only for overall median length of hospitalization.

Decreases in the risks of severe events such as ICU admission and mortality have been estimated from February to June 2020, with corresponding increases in the risks of the positive outcomes of discharge from either ICU or hospital. Total LoS in hospital, averaged over final outcome, has decreased over the same months. Similarly, lengths of stay pre-ICU, in ICU, in hospital overall for those not admitted to ICU, and in post-ICU wards, among survivors who are eventually discharged, have reduced over time. Moreover (Fig. 3), the time to discharge, both from ordinary ward and from post-ICU, is the measure changing most substantially over time. These decreases, altogether, suggest an improvement in patient management, supported by the progressive increase in clinical knowledge of COVID-19 and a less severe disease presentation at hospital admission, resulting from prolonged and strict lockdown measures over the course of 3 months. In contrast, there is less evidence of any change in the lengths of stay for non-survivors and furthermore, the effect of bed capacity on LoS is not significant for non-survivors. This finding may indicate that more frail patients were unfortunately largely impacted by their condition and thus less responsive to the progressive amelioration of care, although no specific cure has been found yet. The long LoS for patients on the pathway hospital-admission-to-ICU-to-PostICU-to-discharge (40.7 days [40.1–41.8]) may be affected by the lack of downstream beds: during the first wave of the pandemic, hospitals struggled to find facilities for post-hospitalization care and rehabilitation. This shortage might have affected discharges from hospitals, as also hypothesized by Boelle et al. [13]. Regione Lombardia has a high proportion of individuals older than 65 (Additional file 1: Appendix A.1.1), including those resident in care homes and long-term facilities, explaining the scarcity of available post-hospital beds. Increasing the number of beds in both long-term facilities and in post-ICU wards where only low-grade assistance is needed, would relieve hospitals and increase patient turnover.

Hospital size appears associated with length of stay, with shorter stays in larger hospitals, at least for survivors, with small but significant effects. This finding remains both when adjusting for month of admission and when adjusting additionally for patient characteristics such as age, gender and co-morbidities [12]. The largest hospitals have more beds, more patients and more skilled staff, and most of them were among the 17 hospitals in the Covid-19 Network. While the association may be confounded with the different case-mix in smaller versus larger hospitals, nevertheless, we posit that our finding reflects the implementation of the hub-and-spoke model: high-risk patients transferred from smaller, less resourced hospitals to specialist, highly skilled hospitals with ICU beds, supporting the “high case volume-better performances” model [15]. The evidence of hospital size effect is very important, as it is a proxy of the effective impact of the emergency measures on hospital management and on patients’ lives, helping inform the management of further pandemic waves.

Some assumptions and limitations to this analysis are inevitable. The 12% of cases without information on their admitting hospital were excluded. The analysis assumes this missingness is ignorable, i.e. that cases with missing hospital information were similar to those with observed hospitals of admission. Similarly, excluding patients with inconsistent data on ICU admissions and discharges is assumed not to have biased results in any way. Hospital bed capacity was defined in terms of a combination of numbers of hospital and ICU beds (see Additional file 1: Appendix Table A.3). Bed capacity could have been defined in different ways, so results could depend on this definition. The fact that a large proportion of hospitals (over 50%) had zero ICU beds may also have caused artefactual results in terms of the risk of ICU admission in small hospitals. Finally, the analysis presented, assuming individuals with missing outcomes are censored 1 day after their last observed event, was demonstrated to be very similar to assuming the missingness is ignorable. The results might not have been as similar if a different censoring assumption had been chosen, such as censoring individuals with missing outcomes at the date of the data (17th July). Such an assumption implies individuals being alive/next event-free for a longer period of time than a single day, so estimates of times to next events would be expected to be larger, although estimated risks would be expected to be similar to those under the 1-day censoring assumption, due to low proportions censored.

Nevertheless, the presented estimates give crucial evidence to support planning hospital care for the current and any potential future wave of infection.

## Supplementary Information

**Additional file 1.**

## Data Availability

The data that support the findings of this study are available from Regione Lombardia but restrictions apply to the availability of these data, which were used under license for the current study, and so are not publicly available. Data are however available from the authors upon reasonable request and with permission of Covid-19 Research Committee of Regione Lombardia.
