# Supplementary material for: Decreasing hospital burden of COVID-19 during the first wave in Regione Lombardia: an emergency measures context
Source: BMC Public Health. 2021 Sep 3;21:1612. doi: 10.1186/s12889-021-11669-w (PMC8414029; doi:10.1186/s12889-021-11669-w)
Supplement: Supplementary file 1 — Additional file 1. [file 12889_2021_11669_MOESM1_ESM.pdf]

Appendix

A.1 Data

A.1.1 Demographic data of Regione Lombardia

Age range	Number of inhabitants
0-18	1,737,444
19-30	1,201,644
31-45	1,964,750
46-55	167,822
56-65	1,335,876
65+	2,186,031

Table A.1: number of inhabitants per age group

The ageing index (defined in Appendix A.4) increased from 145.5 in 2012 to 165.5 in 2019, while the old-age dependency index (defined in Appendix A.4) increased by 3.4% from 2012 to 2019 (1). The most common causes of death in the population are: oncological diseases, cerebrovascular diseases, cardiac ischemic diseases, other cardiac diseases, and circulatory system diseases (2).

The first 10 causes of death among males in Lombardia region: oncological diseases of upper and lower airways, cerebrovascular diseases, ischemic and non-ischemic cardiac diseases, other oncological diseases, acute myocardial

infarction, circulatory system diseases, chronic diseases of lower airways, oncological diseases of lower GI tract, oncological diseases of liver and intrahepatic ducts.

The first 10 causes of death among females in Lombardia region: cerebrovascular diseases, cardiac diseases, circulatory system diseases, cardiac ischemic diseases, oncological diseases of breasts, dementia, other oncological diseases, acute myocardial infarction, oncological disorders of upper and lower airways, Alzheimer's disease.

A.1.2 Emergency Measures

Several pre-pandemic emergency measures were implemented, such as surveillance of in-bound flights from China(3); three laboratories being designated to carry out RT-PCR analysis of nasopharyngeal swabs; and identifying hospitals with at least one infectious diseases ward to admit suspected Covid-19 patients, requiring them and all hospitals in the region to assess their PPE stock (4). In addition, several Regional Units were created:

- a Regional task force (for management of the crisis);
- a Regional Unit of Coordination for Admission to ICU wards;
- a Regional Unit of Coordination for Hospital Admission (to acute wards), that was deputed to the management of available and staffed hospital beds in wards;
- a Regional Unit of Coordination for Hospital Discharge, that was in charge of managing patients discharged from hospitals and providing a recovery facility for further treatment or respiratory rehabilitation in a non-hospital setting. In order to increase consistency among the regional hospitals and for governance purposes, discharge criteria for hospitals to follow were distributed (5), as reported in Table A.2. Eligibility criteria for COVID-19 admission to the recovery facilities were provided (afebrile status for >3 days and improved respiratory symptoms). Integrated home care for patients that could not be transported to hospitals was set to be provided 24 hours per day, every day of the week;
- a Regional Unit of Coordination for Local Health Agencies.

Covid Patients				
Coming from:	Discharge criteria	Exclusion Criteria	Going to:	Type of care:
-Intensive Care Unit<br>-Subintensive Care Unit<br>-Infectious Disease Department<br>-Pneumology Department	-No fever for at least 3 days<br>-PaO2/FiO2 > 250 w/o oxygen support<br>-PaO2/FiO2>300 with oxygen support<br>-NIV or CPAP weaned from at least 72 hrs	-CPAP and NIV ongoing therapy<br>-Continuous O2 flux more than 10 L per minute but RF>20<br>-Dyspnea and VAS>4<br>-Hemodynamic alterations<br>-Severe arrhythmias<br>-Ongoing Fever<br>-Parenteral Nutrition<br>-Bilateral Lung infiltrates<br>-Multiorgan failure	-Subacute Care<br><br>-Respiratory Rehabilitation	-Medical and nursing care 24 hrs<br><br>-Possibility of re-admission to hospital in case of exacerbation or relapse of the disease
-Internal Medicine Department	-patients with comorbidities that have stable parameters<br>- no fever for at least 3 days<br>-Continuous O2 flux less than 4 L per minute		-Socio-medical infrastructure Appointed to Covid 19 patients reception as Nursing Homes	-Medical care available at night<br><br>-Nursing care 24 hrs<br><br>- Possibility of re-admission to hospital in case of exacerbation or relapse of the disease

Table A.2: criteria for discharging Covid-19 patients from hospitals; DGR 2906

A.1.3 Covid-19 Regional Database

The Prevention Unit (Prev) of the General Directorate of Welfare of Regione Lombardia is in charge of monitoring the trends of transmittable diseases in the region. As the SARS-CoV-2 pandemic was breaking out in Lombardia in February 2020, the Prev received Covid-19 related data from different sources, such as hospitals, laboratories and Local Healthcare Agencies, and created a single integrated id-hinged database. An individual who is suspected to have contracted Covid-19 due to onset of symptoms or an epidemiological link is reported by a healthcare worker in the “regional surveillance online monitor”. Each patient is identified through the regional registry office so that no overlap or mismatch might occur. If his oropharyngeal swab analyzed by RT-PCR yields a positive result, the

individual will be assigned an ID and he will enter the Covid-19 Database of Regione Lombardia. Eventual hospital admission will be recorded and matched to his ID as well as hospital discharge or death.

This Covid-19 Regional Database, as extracted on 5th August 2020, consists of 95,777 records, representing 95,354 individuals with confirmed COVID-19 disease covering the period 1st December 2019 to 17th July 2020. The dataset records age, gender, Local Healthcare Agency district of Lombardia, whether the individual has co-morbidities, whether the individual is a healthcare worker or care home resident, whether or not the individual had symptoms, whether or not the individual was hospitalised, and details of the admitting hospital if the individual is a patient. For each individual, dates of symptom onset, positive laboratory test, hospital admission, ICU admission, ICU discharge, hospital discharge, recovery and death are recorded.

Once duplicate records are excluded, 94,945 records remain, with one record per individual. Excluding patients with inconsistent or invalid hospital or ICU admission or discharge dates leaves 94,474 individuals. Restricting attention to patients who were hospitalised and have non-missing hospital admission dates leaves 46,609 individuals.

The dataset is relatively complete for most covariates of interest. Non-missing data on co-morbidities are used to define a co-morbidity flag: existence of at least one co-morbidity corresponds to a flag equal to 1; whereas missing information on co-morbidity corresponds to a flag equal to 0. The symptoms covariate is missing for the majority (64%) of patients, so is not considered further. District is missing for 5.7% of patients, but is not of primary interest in this analysis, so is also not considered further. Admitting hospital is missing for 12.4% of patients, but as hospital size - defined in terms of hospital bed capacity as small, medium or large, depending on numbers of both hospital and ICU beds (Table A.3) - is of interest, a complete-case analysis is considered, assuming the missingness is ignorable. All other covariates are complete, so after excluding the 12.4% of patients with missing hospital, 40,808 individuals are left in the dataset. Month of hospital admission is a key covariate of interest, to understand changes over time. However, very few hospital admissions occurred in January or July in the dataset, so patients admitted in these months are also excluded, leaving a final sample size of 40,550 individuals. Summaries of the covariates for these individuals are given in Table A.4.

			Number of ICU beds

			Small	Medium	Large
			[0,1]	(1,6]	(6,71]
Number of hospital beds	Small	[0,88]	Small	Small	Medium
			52	1	0
	Medium	(88,191]	Small	Medium	Large
			27	18	5
	Large	(191,1063]	Medium	Large	Large
			5	18	27

Table A.3: Definition of hospital bed capacity. Both the total number of beds (rows) and the number of ICU beds (columns) are categorised into small, medium and large by quantiles over the number of hospitals of the respective numbers of beds (33% in each category for total hospital beds, 50% in the small category and 25% in the medium and large categories for ICU beds). These definitions for total and ICU beds are then combined into a single "hospital bed capacity" variable as shown in the table cells, resulting in 80 small hospitals, 23 medium hospitals and 50 large hospitals.

Covariate	Level	Number	Proportion
Age group	[0,45]	4,236	10.45%
	(45,65]	13,010	32.08%
	(65,Inf]	23,304	57.47%
Gender	female	16,063	39.61%
	male	24,487	60.39%
Month of admission	Feb	1,606	3.96%
	Mar	28,101	69.30%
	Apr	8,813	21.73%
	May	1,525	3.76%

	Jun	505	1.25%
**At least 1 co-morbidity?**	no	14,787	36.47%
	yes	25,763	63.53%
**Care home resident?**	no	38,825	95.75%
	yes	1,725	4.25%
**Healthcare worker?**	no	38,889	95.90%
	yes	1,661	4.10%
**Hospital bed capacity**	Large	28,663	70.69%
	Medium	5,288	13.04%
	Small	6,599	16.27%

Table A.4: Covariate summaries

The observed progression of patients through hospital (i.e. raw numbers and proportions transitioning between states) are shown in Figure A.1. From the initial admitting ward, patients progress to either being discharged, being admitted to ICU, or dying. From an ICU ward, patients are either discharged to a post-ICU hospital stay, or die. From a post-ICU stay, patients are either discharged or die. Patients transitioning from either the starting admitting ward state (black box) or an intermediate (white box) state to the "end" node represent individuals with unknown next event (<1% from "ward" and "ICU"; 15% from "post-ICU"). It is unknown whether the missing outcome had not happened by the end-date of the data, 17th July, ("right-censoring"), or whether the final outcome had happened, but was not recorded ("missing data/loss to follow-up").

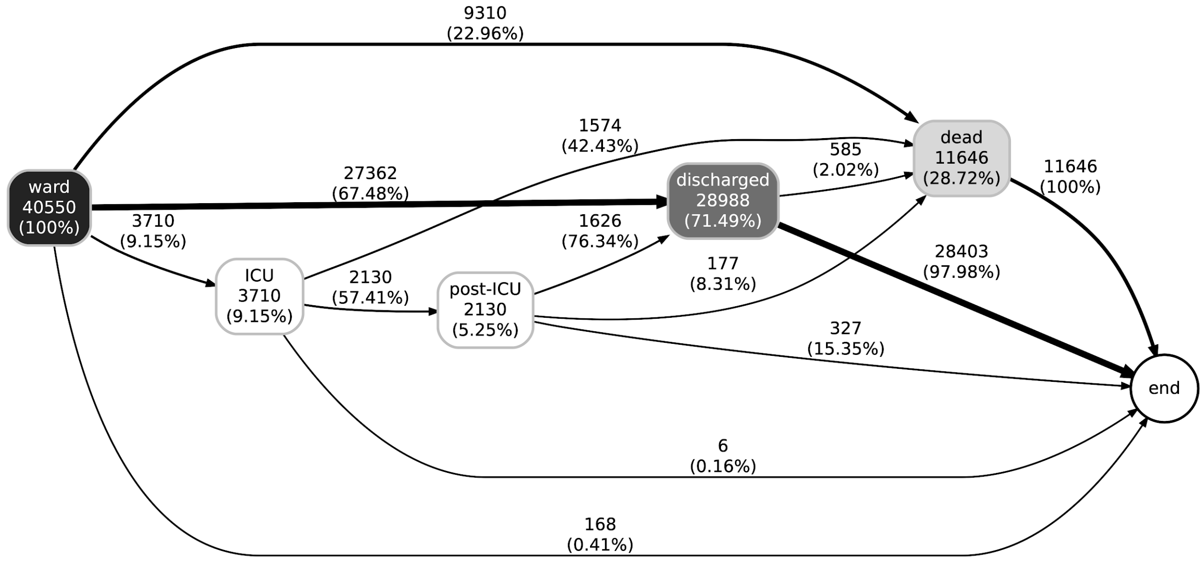

Figure A.1: Observed progression through hospital to final outcomes. Patients start in the hospital admission to a ward state (black box). The number (proportion) in each state (white and grey boxes) represents the total number (proportion) who reach each state, from any previous state: white boxes are the intermediate states of admittance to ICU and a post-ICU hospital stay; the grey boxes are the "absorbing" states representing the final outcomes of death or discharge. Numbers (proportions) along each arrow represent the number of patients transitioning from one state to the next.

A.2 Mixture competing risks multi-state model

Jackson et al (7) extended the mixture competing risks framework of Larson & Dinse (11), which considered competing risks of transitions to next events from a single state, to a general multi-state model. Each individual *i* in state *r* makes a transition to a next event *s* at time *t* according to a transition intensity

$$\lambda_{i,r,s}(t) \ = \lambda^*{}_{i,r,s}(t) \ \text{ if } I_{i,r} = s, \text{ or 0 otherwise}$$

where $I_{i,r}$ is a latent categorical variable determining which event happens next. The next event is governed by probabilities $\pi_{r,s} = P(I_{i,r} = s)$ where these probabilities sum to 1 over all next events *s* possible from starting state *r*. The transition intensity $\lambda^*{}_{i,r,s}(t)$ is defined by the hazard function of a parametric distribution that governs the time $T_{r,s}$ from entering state *r* until the next event *s*, conditional on the *r*-*s* transition being the one that occurs.

We consider different parametric forms for the times $T_{r,s} \sim f(T_{r,s})$, choosing between gamma, Weibull, log-normal and generalised gamma distributions according to likelihood ratio tests (the gamma, Weibull and log-normal distributions are special cases of the generalised gamma distribution) minimum AIC.

When considering covariates, either binomial or multinomial logistic regression is used for the probabilities $\pi_{r,s}$, and the location (mean) parameter of the selected time-to-next-event distribution is regressed on the covariates. For the probabilities, covariate effects β correspond to odds ratios $exp(\beta)$ for each covariate level relative to their baseline. For the times, covariate effects γ correspond to "expected time ratios" $exp(\gamma)$ which act multiplicatively on the expected time from the current state to the next event.

The multi-state model describing in-hospital progression is displayed in Figure A.2.

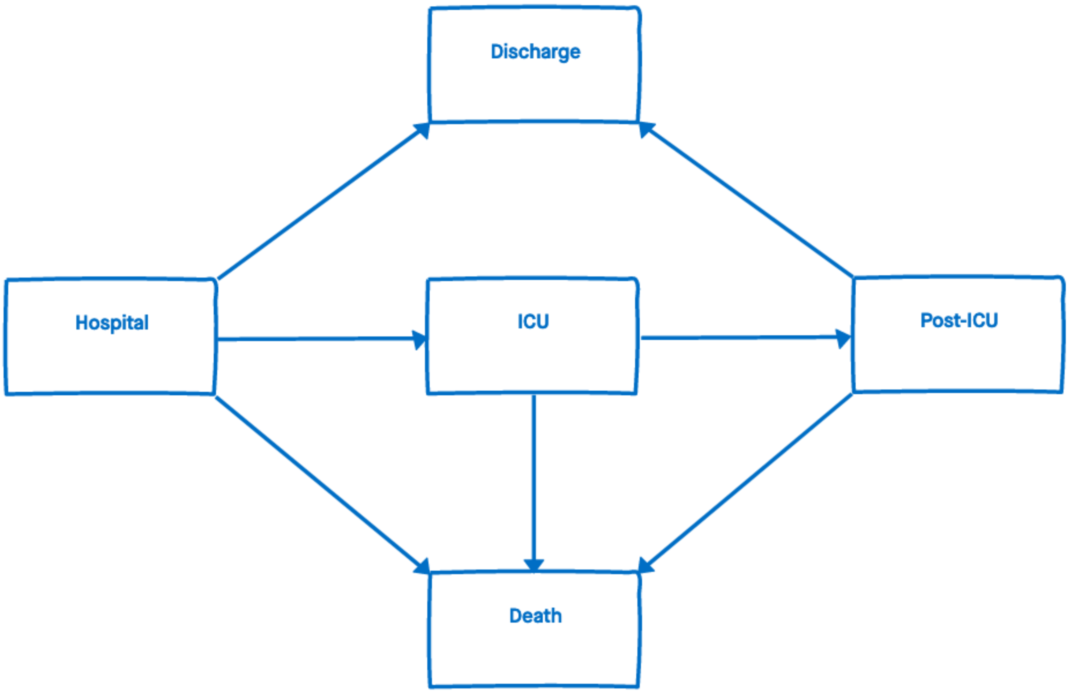

Figure A.2: Multi-state model

A.3 Detailed results

A.3.1 Model with no covariates

Parameter estimates from this model are given in Table A.5:

				Estimate		SE	
**From**	**To**	**Distribution**	**Parameter**	**Censoring**	**Missing**	**Censoring**	**Missing**
Hospital	Discharge		$\pi_{1,2}$	0.6776	0.6776	0.0203	0.0203
Hospital	ICU		$\pi_{1,3}$	0.0918	0.0919	0.0174	0.0175
Hospital	Death		$\pi_{1,5}$	0.2306	0.2305	0.0119	0.0120
Hospital	Discharge	gamma	shape	0.7801	0.7794	0.0074	0.0074
Hospital	Discharge	gamma	rate	0.0452	0.0452	0.0101	0.0101
Hospital	ICU	gamma	shape	0.7978	0.7968	0.0200	0.0200
Hospital	ICU	gamma	rate	0.1422	0.1421	0.0272	0.0272
Hospital	Death	gamma	shape	1.1015	1.1010	0.0130	0.0130
Hospital	Death	gamma	rate	0.1159	0.1159	0.0163	0.0163
ICU	Post-ICU		$\pi_{3,4}$	0.5751	0.5751	0.0332	0.0332
ICU	Death		$\pi_{3,5}$	0.4249	0.4249	0.0332	0.0332
ICU	Post-ICU	gamma	shape	1.2785	1.2788	0.0275	0.0276
ICU	Post-ICU	gamma	rate	0.0765	0.0765	0.0336	0.0336
ICU	Death	gamma	shape	1.4917	1.4918	0.0324	0.0324
ICU	Death	gamma	rate	0.1220	0.1220	0.0384	0.0384
Post-ICU	Discharge		$\pi_{4,2}$	0.9019	0.9018	0.0679	0.0791
Post-ICU	Death		$\pi_{4,5}$	0.0981	0.0982	0.0679	0.0791
Post-ICU	Discharge	gamma	shape	2.1014	2.0990	0.0325	0.0327
Post-ICU	Discharge	gamma	rate	0.0946	0.0945	0.0368	0.0369

Post-ICU	Death	gamma	shape	1.6067	1.5849	0.0953	0.0970
Post-ICU	Death	gamma	rate	0.1281	0.1265	0.1127	0.1139

Table A.5: parameter estimates for the model with no covariates, by missing outcome assumption: (a) censoring at 1 day after last observed event; (b) ignoring missing outcomes.

Comparison of non-parametric and parametric cumulative incidence curves, under both the missing outcome assumptions, are shown in Figure A.3:

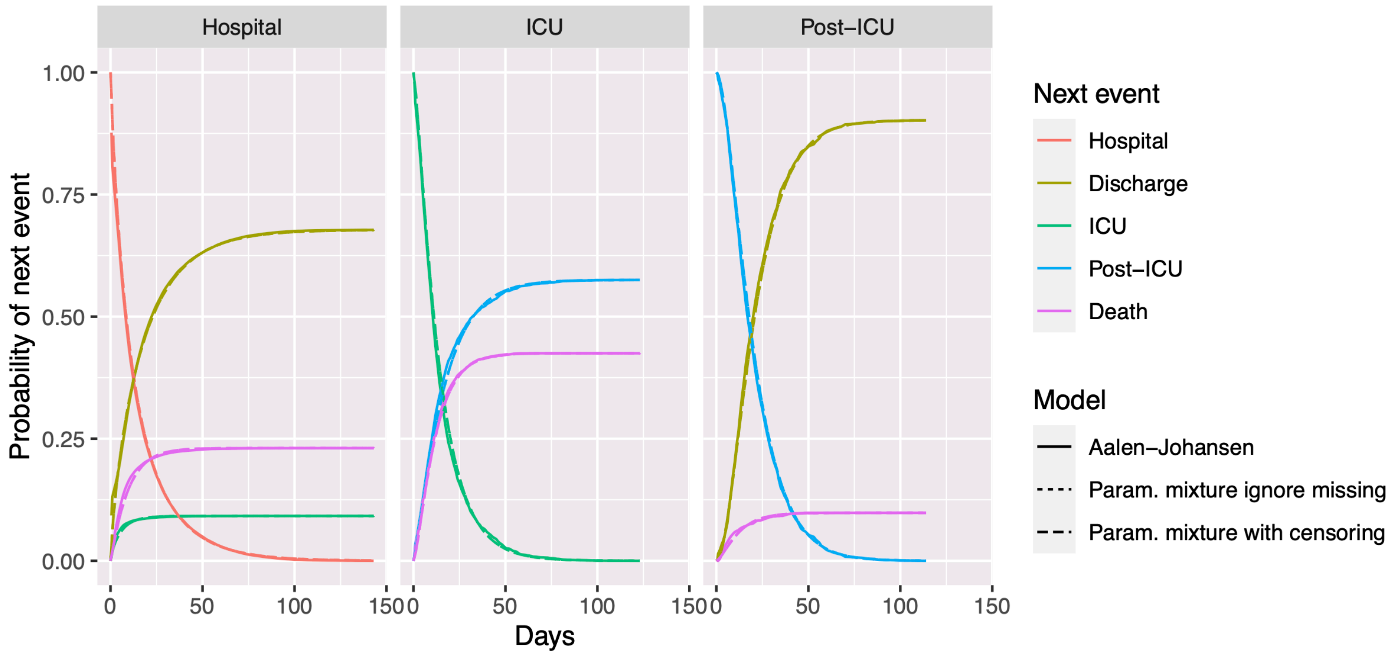

Figure A.3: Parametric versus non-parametric cumulative incidence estimates, by starting state (columns), next event (colours) and model (line type).

Estimated probabilities of next events are given in Table A.6:

		CENSORING			MISSING		
**From**	**Next Event**	**Pr(Next Event)**	**Lower**	**Upper**	**Pr(Next Event)**	**Lower**	**Upper**
Hospital	Discharge	0.678	0.673	0.681	0.678	0.674	0.681
	ICU	0.092	0.090	0.095	0.092	0.090	0.094

	Death	0.231	0.227	0.235	0.231	0.227	0.235
ICU	Post-ICU	0.575	0.561	0.592	0.575	0.556	0.586
	Death	0.425	0.408	0.439	0.425	0.414	0.444
Post-ICU	Discharge	0.902	0.890	0.910	0.902	0.889	0.912
	Death	0.098	0.090	0.110	0.098	0.088	0.111

Table A.6: Estimated probabilities (95% confidence intervals) of next events, given current state, by missing outcome assumption: (a) censoring at 1 day after last observed event; (b) ignoring missing outcomes.

Estimated probabilities of final events are given in Table A.7:

	**CENSORING**			**MISSING**		
**Final Event**	**Pr(Final Event)**	**Lower**	**Upper**	**Pr(Final Event)**	**Lower**	**Upper**
Death	0.275	0.271	0.280	0.275	0.271	0.278
Discharge	0.725	0.720	0.729	0.725	0.722	0.729

Table A.7: Estimated probabilities (95% confidence intervals) of final events, given current state (hospital-fatality risks and complement), by missing outcome assumption: (a) censoring at 1 day after last observed event; (b) ignoring missing outcomes.

Estimated times to next events, under the censoring assumption only, are given in Table A.8:

**From**	**Next Event**	**Mean**	**95% CI of Mean**		**Median**	**95% CI of Median**		**25%-ile**	**95% CI of 25%-ile**		**75%-ile**	**95% CI of 75%-ile**	
Hospital	Discharge	17.3	17.0	17.6	10.7	10.6	10.8	3.7	3.7	3.8	23.8	23.6	24.1
Hospital	ICU	5.6	5.4	5.8	3.5	3.3	3.6	1.2	1.2	1.3	7.8	7.4	8.0
Hospital	Death	9.5	9.2	9.6	6.8	6.7	7.0	3.0	2.9	3.1	13.2	13.0	13.4
ICU	Post-ICU	16.7	16.2	17.2	12.6	12.2	13.2	6.0	5.8	6.4	23.1	22.3	24.1
ICU	Death	12.2	11.8	12.6	9.6	9.3	10.1	4.9	4.7	5.2	16.8	16.1	17.4

Post-ICU	Discharge	22.2	21.5	22.9	18.8	18.2	19.5	11.0	10.4	11.6	29.8	28.9	30.8
Post-ICU	Death	12.5	10.8	13.7	10.1	8.9	11.9	5.3	4.5	6.6	17.1	15.1	19.5

Table A.8: Summaries of times from current state to next event, conditional on experiencing that next event, assuming missing outcomes are censoring at 1 day after last observed event.

Estimated times to final events (total length of stay in hospital), by pathway through hospital, are given in Table A.9, under the censoring assumption only:

**Outcome**	**Pathway**	**Mean**	**95% CI of Mean**		**Median**	**95% CI of Median**		**25%-ile**	**95% CI of 25%-ile**		**75%-ile**	**95% CI of 75%-ile**	
Death	Hospital-Death	9.5	9.3	9.6	6.9	6.6	7.1	3.0	2.9	3.1	13.2	12.8	13.5
Death	Hospital-ICU-Death	17.8	17.4	18.4	15.1	14.8	15.9	9.1	8.7	9.6	23.7	23.1	24.5
Death	Hospital-ICU-Post-ICU-Death	34.9	33.7	36.4	31.7	30.0	33.0	21.2	20.0	22.5	44.6	42.8	47.1
Discharge	Hospital-Discharge	17.3	17.1	17.5	10.8	10.3	10.9	3.7	3.5	3.9	23.9	23.1	24.6
Discharge	Hospital-ICU-Post-ICU-Discharge	44.6	43.7	45.4	40.7	40.1	41.8	28.1	28.0	29.0	56.5	55.8	58.3
Death	Averaged over pathways	11.2	11.0	11.4	8.1	7.8	8.5	3.6	3.4	3.7	15.2	15.0	16.0
Discharge	Averaged over pathways	19.1	18.8	19.3	11.8	11.6	12.3	4.0	3.9	4.3	27.1	26.0	27.8

Table A.9: Summaries of times from hospital admission to final events (total length of stay), by pathway through hospital, conditional on experiencing that final event, assuming missing outcomes are censoring at 1 day after last observed event.

Estimated lengths of stay in hospital, ICU and post-ICU, averaged over pathways and final outcomes, are given in Table A.10, under the censoring assumption only:

	**Mean**	**95% CI of Mean**	**Median**	**95% CI of Median**	**25%-ile**	**95% CI of 25%-ile**	**75%-ile**	**95% CI of 75%-ile**

Hospital	16.9	16.7	17.0	10.4	10.1	10.9	3.8	3.8	4.1	22.6	22.5	23.8
ICU	14.8	14.3	15.1	11.0	10.7	11.6	5.6	5.2	5.7	19.7	19.5	20.9
Post-ICU	21.3	20.6	21.8	18.0	17.1	18.7	10.1	9.6	10.5	28.8	27.5	30.1

Table A.10: Summaries of lengths of stay in hospital (total time in hospital), ICU and post-ICU wards, averaged over pathways and final outcomes, assuming missing outcomes are censoring at 1 day after last observed event.

A.3.2 Model regressed on month of admission

Parameter estimates from this model are given in Table A.11:

				**Estimate**		**SE**		**OR or ETR**		**Lower**		**Upper**	
**From**	**To**	**Distribution**	**Parameter**	**Censor**	**Missing**	**Censor**	**Missing**	**Censor**	**Missing**	**Censor**	**Missing**	**Censor**	**Missing**
Hospital	Discharge		$\pi_{1,2}$	0.6487	0.6486	0.0232	0.0232						
Hospital	ICU		$\pi_{1,3}$	0.1045	0.1045	0.0199	0.0199						
Hospital	Death		$\pi_{1,5}$	0.2469	0.2469	0.0141	0.0141						
Hospital	Discharge		$\pi_{1,2}$Feb	0.3890	0.3929	0.0761	0.0761	1.4755	1.4813	1.2711	1.2761	1.7127	1.7195
Hospital	Discharge		$\pi_{1,2}$Apr	-0.7601	-0.7589	0.0505	0.0508	0.4676	0.4682	0.4235	0.4238	0.5163	0.5172
Hospital	Discharge		$\pi_{1,2}$May	-1.4800	-1.4805	0.1497	0.1513	0.2276	0.2275	0.1697	0.1691	0.3053	0.3060
Hospital	Discharge		$\pi_{1,2}$Jun	-1.7210	-1.7226	0.2680	0.2938	0.1789	0.1786	0.1058	0.1004	0.3025	0.3177
Hospital	ICU		$\pi_{1,3}$Feb	0.1182	0.1183	0.0604	0.0606	1.1255	1.1256	0.9998	0.9997	1.2670	1.2675
Hospital	ICU		$\pi_{1,3}$Apr	-0.3578	-0.3575	0.0303	0.0305	0.6992	0.6994	0.6589	0.6589	0.7420	0.7425
Hospital	ICU		$\pi_{1,3}$May	-0.8463	-0.8438	0.0758	0.0764	0.4290	0.4301	0.3698	0.3703	0.4977	0.4995
Hospital	ICU		$\pi_{1,3}$Jun	-1.6530	-1.6620	0.1717	0.1894	0.1915	0.1897	0.1368	0.1309	0.2681	0.2751

Hospital	Discharge	gengamma	$\mu_{1,2}$	2.9678	2.9679	0.0134	0.0134						
Hospital	Discharge	gengamma	$\sigma_{1,2}$	1.0468	1.0472	0.0072	0.0072						
Hospital	Discharge	gengamma	$Q_{1,2}$	1.3271	1.3284	0.0197	0.0198						
Hospital	Discharge	gengamma	$T_{1,2}$Feb	0.6777	0.6773	0.0347	0.0347	1.9694	1.9686	1.8399	1.8392	2.1079	2.1071
Hospital	Discharge	gengamma	$T_{1,2}$Apr	-0.0425	-0.0426	0.0151	0.0151	0.9584	0.9583	0.9304	0.9304	0.9872	0.9871
Hospital	Discharge	gengamma	$T_{1,2}$May	-0.3932	-0.3934	0.0305	0.0305	0.6749	0.6748	0.6356	0.6356	0.7165	0.7163
Hospital	Discharge	gengamma	$T_{1,2}$Jun	-0.8019	-0.8058	0.0518	0.0518	0.4485	0.4467	0.4052	0.4036	0.4963	0.4945
Hospital	ICU	gengamma	$\mu_{1,3}$	0.8490	0.8496	0.0439	0.0432						
Hospital	ICU	gengamma	$\sigma_{1,3}$	1.2354	1.2359	0.0125	0.0125						
Hospital	ICU	gengamma	$Q_{1,3}$	-0.2507	-0.2492	0.0600	0.0588						
Hospital	ICU	gengamma	$T_{1,3}$Feb	0.0940	0.0935	0.0851	0.0851	1.0985	1.0981	0.9298	0.9294	1.2979	1.2973
Hospital	ICU	gengamma	$T_{1,3}$Apr	-0.1716	-0.1710	0.0604	0.0603	0.8423	0.8428	0.7483	0.7488	0.9482	0.9486
Hospital	ICU	gengamma	$T_{1,3}$May	-0.4879	-0.4938	0.1833	0.1824	0.6139	0.6103	0.4286	0.4269	0.8794	0.8725
Hospital	ICU	gengamma	$T_{1,3}$Jun	-0.9796	-1.0859	0.3486	0.3481	0.3755	0.3376	0.1896	0.1707	0.7436	0.6679
Hospital	Death	gengamma	$\mu_{1,5}$	1.8330	1.8330	0.0183	0.0183						
Hospital	Death	gengamma	$\sigma_{1,5}$	1.0108	1.0108	0.0079	0.0079						
Hospital	Death	gengamma	$Q_{1,5}$	0.3674	0.3681	0.0271	0.0271						
Hospital	Death	gengamma	$T_{1,5}$Feb	1.1846	1.1863	0.0514	0.0513	3.2695	3.2751	2.9564	2.9616	3.6157	3.6217
Hospital	Death	gengamma	$T_{1,5}$Apr	0.1861	0.1865	0.0272	0.0272	1.2045	1.2051	1.1419	1.1425	1.2705	1.2710
Hospital	Death	gengamma	$T_{1,5}$May	0.1478	0.1488	0.0716	0.0716	1.1593	1.1604	1.0075	1.0084	1.3340	1.3353
Hospital	Death	gengamma	$T_{1,5}$Jun	-0.2632	-0.2797	0.1832	0.1867	0.7686	0.7560	0.5367	0.5243	1.1007	1.0900
ICU	Post-ICU		$\pi_{3,4}$	0.5586	0.5582	0.0371	0.0372						
ICU	Death		$\pi_{3,5}$	0.4414	0.4418	0.0371	0.0372						
ICU	Post-ICU		$\pi_{3,4}$Feb	0.0745	0.0676	0.1375	0.1377	1.0773	1.0699	0.8228	0.8168	1.4107	1.4015
ICU	Post-ICU		$\pi_{3,4}$Apr	-0.4760	-0.4839	0.1024	0.1034	0.6213	0.6164	0.5083	0.5033	0.7594	0.7548
ICU	Post-ICU		$\pi_{3,4}$MayJun	-0.8046	-0.8192	0.3009	0.3022	0.4473	0.4408	0.2480	0.2438	0.8067	0.7969

ICU	Post-ICU	gengamma	$\mu_{3,4}$	2.7774	2.7775	0.0339	0.0339						
ICU	Post-ICU	gengamma	$\sigma_{3,4}$	0.9166	0.9166	0.0185	0.0185						
ICU	Post-ICU	gengamma	$Q_{3,4}$	0.7055	0.7057	0.0537	0.0537						
ICU	Post-ICU	gengamma	$T_{3,4}$Feb	-0.2137	-0.2137	0.0853	0.0857	0.8076	0.8076	0.6832	0.6828	0.9546	0.9553
ICU	Post-ICU	gengamma	$T_{3,4}$Apr	-0.1802	-0.1802	0.0553	0.0553	0.8351	0.8351	0.7493	0.7493	0.9308	0.9307
ICU	Post-ICU	gengamma	$T_{3,4}$MayJun	-0.5303	-0.5303	0.1182	0.1200	0.5884	0.5884	0.4668	0.4651	0.7417	0.7444
ICU	Death	gengamma	$\mu_{3,5}$	2.4975	2.4975	0.0353	0.0353						
ICU	Death	gengamma	$\sigma_{3,5}$	0.8077	0.8077	0.0227	0.0227						
ICU	Death	gengamma	$Q_{3,5}$	0.8434	0.8436	0.0631	0.0632						
ICU	Death	gengamma	$T_{3,5}$Feb	0.1253	0.1253	0.0819	0.0819	1.1334	1.1335	0.9654	0.9655	1.3308	1.3307
ICU	Death	gengamma	$T_{3,5}$Apr	0.1524	0.1524	0.0677	0.0677	1.1646	1.1646	1.0198	1.0199	1.3300	1.3297
ICU	Death	gengamma	$T_{3,5}$MayJun	-0.9603	-0.9597	0.2091	0.2088	0.3828	0.3830	0.2541	0.2544	0.5767	0.5767
Post-ICU	Discharge		$\pi_{4,2}$	0.9158	0.9158	0.0831	0.0966						
Post-ICU	Death		$\pi_{4,5}$	0.0842	0.0842	0.0831	0.0966						
Post-ICU	Discharge		$\pi_{4,2}$Feb	1.5685	1.5686	0.1957	0.2395	4.7996	4.8002	3.2708	3.0017	7.0429	7.6761
Post-ICU	Discharge		$\pi_{4,2}$Apr	0.0567	0.0568	0.2000	0.2307	1.0583	1.0584	0.7152	0.6734	1.5662	1.6635
Post-ICU	Discharge		$\pi_{4,2}$MayJun	0.6623	0.6642	0.3922	0.4950	1.9392	1.9429	0.8990	0.7364	4.1830	5.1266
Post-ICU	Discharge	gengamma	$\mu_{4,2}$	3.1393	3.1403	0.0277	0.0277						
Post-ICU	Discharge	gengamma	$\sigma_{4,2}$	0.6809	0.6810	0.0206	0.0206						
Post-ICU	Discharge	gengamma	$Q_{4,2}$	0.7143	0.7180	0.0563	0.0566						
Post-ICU	Discharge	gengamma	$T_{4,2}$Feb	0.0185	0.0186	0.0847	0.0849	1.0186	1.0188	0.8627	0.8627	1.2027	1.2032
Post-ICU	Discharge	gengamma	$T_{4,2}$Apr	-0.1410	-0.1411	0.0465	0.0465	0.8684	0.8684	0.7927	0.7927	0.9514	0.9513
Post-ICU	Discharge	gengamma	$T_{4,2}$MayJun	-0.5581	-0.5587	0.1297	0.1299	0.5723	0.5720	0.4438	0.4434	0.7379	0.7378
Post-ICU	Death	gengamma	$\mu_{4,5}$	2.1023	2.1032	0.1460	0.1473						
Post-ICU	Death	gengamma	$\sigma_{4,5}$	0.8267	0.8269	0.0569	0.0570						
Post-ICU	Death	gengamma	$Q_{4,5}$	-0.2461	-0.2443	0.2665	0.2696						

Post-ICU	Death	gengamma	$T_{4,5}$Feb	-0.1557	-0.1561	0.1728	0.1728	0.8558	0.8555	0.6100	0.6098	1.2008	1.2002
Post-ICU	Death	gengamma	$T_{4,5}$Apr	0.0548	0.0545	0.1852	0.1854	1.0563	1.0560	0.7348	0.7343	1.5185	1.5186
Post-ICU	Death	gengamma	$T_{4,5}$MayJun	-0.1854	-0.1863	0.3842	0.3863	0.8307	0.8301	0.3912	0.3893	1.7639	1.7699

Table A.11: parameter estimates for the model regressed on month of admission, by missing outcome assumption: (a) censoring at 1 day after last observed event; (b) ignoring missing outcomes. OR refers to odds ratios for the probabilities of each transition in each month relative to March. ETR refers to the expected time ratios for the times of each transition in each month relative to March.

Comparison of non-parametric and parametric cumulative incidence curves, under both the missing outcome assumptions, are shown in Figure A.4:

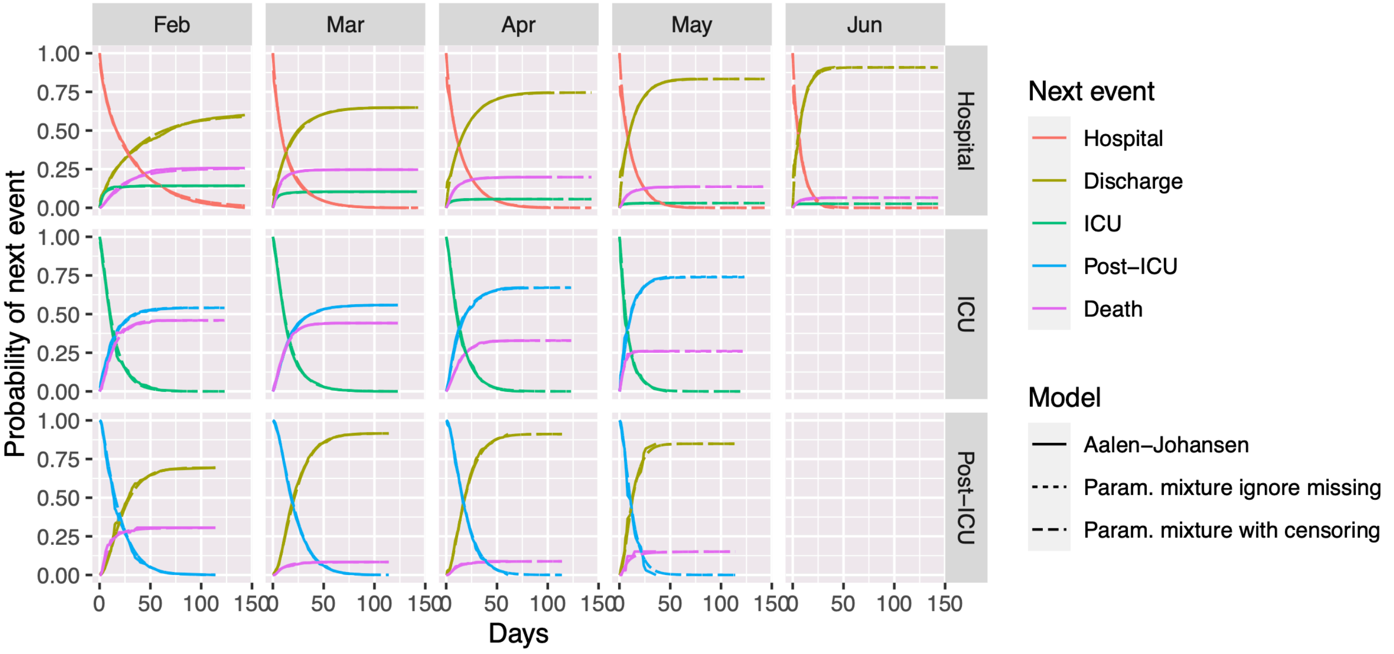

Figure A.4: Parametric versus non-parametric cumulative incidence estimates, by starting state (rows), next event (colours), month of admission (columns) and model (line type). Note that for the ICU and post-ICU states, due to small sample sizes, May and June are combined and shown in the May column.

Estimated probabilities of next events are given in Table A.12:

			**CENSORING**	**MISSING**

From	Next Event	Month	Pr(Next Event)	Lower	Upper	Pr(Next Event)	Lower	Upper
Hospital	Discharge	Feb	0.600	0.580	0.620	0.600	0.582	0.623
Hospital	Discharge	Mar	0.649	0.644	0.655	0.649	0.643	0.654
Hospital	Discharge	Apr	0.745	0.734	0.754	0.745	0.736	0.754
Hospital	Discharge	May	0.833	0.809	0.850	0.833	0.816	0.846
Hospital	Discharge	Jun	0.908	0.894	0.929	0.908	0.886	0.927
Hospital	ICU	Feb	0.143	0.130	0.160	0.143	0.128	0.157
Hospital	ICU	Mar	0.104	0.100	0.107	0.105	0.101	0.108
Hospital	ICU	Apr	0.056	0.053	0.060	0.056	0.052	0.061
Hospital	ICU	May	0.031	0.023	0.039	0.031	0.024	0.042
Hospital	ICU	Jun	0.026	0.017	0.036	0.026	0.019	0.047
Hospital	Death	Feb	0.257	0.241	0.276	0.257	0.238	0.272
Hospital	Death	Mar	0.247	0.243	0.251	0.247	0.242	0.251
Hospital	Death	Apr	0.198	0.190	0.208	0.198	0.192	0.205
Hospital	Death	May	0.136	0.119	0.154	0.136	0.125	0.150
Hospital	Death	Jun	0.066	0.050	0.080	0.066	0.047	0.091
ICU	Post-ICU	Feb	0.540	0.472	0.585	0.541	0.480	0.584
ICU	Post-ICU	Mar	0.559	0.541	0.578	0.558	0.543	0.567
ICU	Post-ICU	Apr	0.671	0.634	0.701	0.672	0.643	0.713
ICU	Post-ICU	MayJun	0.739	0.636	0.824	0.741	0.637	0.831
ICU	Death	Feb	0.460	0.415	0.528	0.459	0.416	0.520
ICU	Death	Mar	0.441	0.422	0.459	0.442	0.433	0.457
ICU	Death	Apr	0.329	0.299	0.366	0.328	0.287	0.357
ICU	Death	MayJun	0.261	0.176	0.364	0.259	0.169	0.363
Post-ICU	Discharge	Feb	0.694	0.624	0.765	0.694	0.585	0.791
Post-ICU	Discharge	Mar	0.916	0.903	0.924	0.916	0.897	0.927

Post-ICU	Discharge	Apr	0.911	0.874	0.933	0.911	0.892	0.937
Post-ICU	Discharge	MayJun	0.849	0.701	0.922	0.848	0.742	0.930
Post-ICU	Death	Feb	0.306	0.235	0.376	0.306	0.209	0.415
Post-ICU	Death	Mar	0.084	0.076	0.097	0.084	0.073	0.103
Post-ICU	Death	Apr	0.089	0.067	0.126	0.089	0.063	0.108
Post-ICU	Death	MayJun	0.151	0.078	0.299	0.152	0.070	0.258

Table A.12: Estimated probabilities (95% confidence intervals) of next events, given current state, by month of admission and missing outcome assumption: (a) censoring at 1 day after last observed event; (b) ignoring missing outcomes.

Estimated probabilities of final events are given in Table A.13:

		CENSORING			MISSING		
**Final Event**	**Month**	**Pr(Final Event)**	**Lower**	**Upper**	**Pr(Final Event)**	**Lower**	**Upper**
Death	Feb	0.346	0.325	0.366	0.346	0.329	0.368
Death	Mar	0.298	0.293	0.303	0.298	0.293	0.303
Death	Apr	0.220	0.211	0.230	0.220	0.210	0.228
Death	May	0.147	0.133	0.159	0.148	0.128	0.167
Death	Jun	0.076	0.063	0.106	0.075	0.058	0.104
Discharge	Feb	0.654	0.634	0.675	0.654	0.632	0.671
Discharge	Mar	0.702	0.697	0.707	0.702	0.697	0.707
Discharge	Apr	0.780	0.770	0.789	0.780	0.772	0.790
Discharge	May	0.853	0.841	0.867	0.852	0.833	0.872
Discharge	Jun	0.924	0.894	0.937	0.925	0.896	0.942

Table A.13: Estimated probabilities (95% confidence intervals) of final events, given current state (hospital-fatality risks and complement), by month of admission and missing outcome assumption: (a) censoring at 1 day after last observed event; (b) ignoring missing outcomes.

Estimated times to next events, under the censoring assumption only, are given in Table A.14:

From	Next Event	Month	Mean	95% CI of Mean		Median	95% CI of Median		25%-ile	95% CI of 25%-ile		75%-ile	95% CI of 75%-ile	
Hospital	Discharge	Feb	34.0	31.4	35.5	22.4	21.1	23.6	7.7	7.3	8.2	48.5	46.0	51.1
	Discharge	Mar	17.3	17.0	17.5	11.3	11.2	11.6	3.9	3.8	4.0	24.6	24.3	25.0
	Discharge	Apr	16.5	16.1	16.9	10.9	10.6	11.2	3.7	3.6	3.9	23.6	23.0	24.2
	Discharge	May	11.6	11.1	12.2	7.7	7.2	8.0	2.6	2.5	2.8	16.6	15.6	17.3
	Discharge	Jun	7.7	6.9	8.6	5.1	4.7	5.6	1.8	1.6	1.9	11.0	10.3	12.1
	ICU	Feb	7.3	6.0	8.7	2.8	2.5	3.3	1.3	1.1	1.4	6.8	5.9	7.9
	ICU	Mar	6.6	6.0	7.1	2.6	2.5	2.7	1.1	1.1	1.2	6.2	5.9	6.6
	ICU	Apr	5.6	5.2	6.5	2.2	2.0	2.4	1.0	0.9	1.1	5.2	4.7	5.7
	ICU	May	4.1	3.2	6.2	1.6	1.1	2.3	0.7	0.5	1.0	3.8	2.7	5.6
	ICU	Jun	2.5	1.1	4.3	1.0	0.6	1.6	0.4	0.3	0.7	2.3	1.5	3.7
	Death	Feb	27.5	25.1	30.0	18.0	16.4	19.8	8.7	8.0	9.5	35.1	32.2	38.6
	Death	Mar	8.4	8.2	8.6	5.5	5.4	5.6	2.7	2.6	2.7	10.7	10.5	11.0
	Death	Apr	10.1	9.6	10.4	6.6	6.4	6.9	3.2	3.1	3.4	12.9	12.3	13.5
	Death	May	9.7	8.3	11.5	6.4	5.9	7.3	3.1	2.8	3.6	12.5	11.5	14.2
	Death	Jun	6.5	4.9	9.1	4.2	2.9	5.8	2.1	1.4	2.8	8.3	5.7	11.3
ICU	Post-ICU	Feb	14.1	11.9	17.1	10.3	9.1	13.0	5.0	4.3	6.4	19.1	16.9	24.0
	Post-ICU	Mar	17.5	16.9	18.4	12.8	12.3	13.4	6.2	5.9	6.5	23.7	22.6	24.6
	Post-ICU	Apr	14.6	13.4	16.4	10.7	10.0	12.1	5.2	4.8	5.9	19.8	18.3	22.6
	Post-ICU	MayJun	10.3	8.8	12.2	7.5	6.6	9.7	3.7	3.2	4.7	13.9	12.1	18.0
	Death	Feb	13.6	12.5	15.4	10.8	9.6	12.0	5.5	5.0	6.1	18.7	16.6	20.7
	Death	Mar	12.0	11.6	12.6	9.5	9.2	10.1	4.9	4.6	5.3	16.5	16.0	17.2
	Death	Apr	14.0	12.3	15.8	11.1	9.9	12.3	5.7	5.2	6.4	19.2	17.1	21.5
	Death	MayJun	4.6	3.4	6.8	3.6	2.5	5.3	1.9	1.3	2.7	6.3	4.3	9.1

Post-ICU	Discharge	Feb	23.3	19.5	25.4	19.8	17.7	23.0	11.6	10.3	13.3	31.3	27.9	36.1
	Discharge	Mar	22.8	22.2	23.6	19.5	18.6	20.4	11.4	10.9	12.0	30.7	29.2	31.8
	Discharge	Apr	19.8	18.7	21.5	16.9	15.9	18.3	9.9	9.2	10.7	26.7	25.0	28.9
	Discharge	MayJun	13.1	10.9	17.3	11.1	7.9	13.5	6.5	4.6	7.9	17.6	12.6	21.0
	Death	Feb	11.3	8.0	16.0	7.5	5.7	9.9	4.3	3.2	5.5	13.4	9.9	18.5
	Death	Mar	13.3	10.8	17.7	8.8	7.5	10.1	5.1	4.4	5.8	15.6	14.2	18.2
	Death	Apr	14.0	10.0	22.4	9.3	6.7	12.0	5.4	3.9	7.0	16.5	11.9	22.8
	Death	MayJun	11.0	5.0	24.0	7.3	3.3	15.5	4.2	1.7	9.0	13.0	5.9	27.9

Table A.14: Summaries of times from current state to next event, conditional on experiencing that next event, by month of admission, assuming missing outcomes are censoring at 1 day after last observed event.

Estimated times to final events (total length of stay in hospital), by pathway through hospital, are given in Table A.15, under the censoring assumption only:

**Outcome**	**Pathway**	**Month**	**Mean**	**95% CI of Mean**		**Median**	**95% CI of Median**		**25%-ile**	**95% CI of 25%-ile**		**75%-ile**	**95% CI of 75%-ile**	
Death	Hospital-Death	Feb	27.5	24.9	29.9	18.2	16.3	19.7	9.1	8.1	9.5	34.7	31.6	38.3
Death	Hospital-Death	Mar	8.4	8.2	8.6	5.5	5.3	5.7	2.7	2.5	2.8	10.7	10.4	11.1
Death	Hospital-Death	Apr	10.1	9.8	10.6	6.7	6.4	7.0	3.3	3.1	3.4	13.0	12.4	13.6
Death	Hospital-Death	May	9.7	8.3	11.0	6.4	5.7	7.5	3.1	2.7	3.6	12.4	11.0	14.7
Death	Hospital-Death	Jun	6.5	4.7	8.3	4.2	3.1	5.6	2.1	1.5	2.7	8.3	6.0	10.7
Death	Hospital-ICU-Death	Feb	20.9	19.1	23.0	16.0	14.5	17.9	9.6	8.5	10.5	26.1	23.3	29.5
Death	Hospital-ICU-Death	Mar	18.6	17.9	19.5	14.5	13.8	15.0	8.5	8.0	8.8	23.6	22.3	24.1
Death	Hospital-ICU-Death	Apr	19.6	17.4	21.6	15.4	13.9	16.4	9.0	8.1	9.6	25.0	22.5	26.5
Death	Hospital-ICU-Death	May	8.7	6.6	11.2	6.5	4.7	8.4	3.8	2.8	5.0	10.4	7.6	13.5
Death	Hospital-ICU-Death	Jun	7.1	5.7	9.2	5.5	3.7	7.9	3.3	2.2	4.6	9.0	6.0	12.7
Death	Hospital-ICU-Post-ICU-Death	Feb	32.8	30.4	38.7	26.6	23.3	29.5	17.0	15.2	19.3	40.3	35.5	44.7

Death	Hospital-ICU-Post-ICU-Death	Mar	37.4	34.8	42.1	30.8	28.7	32.5	20.0	18.5	21.3	47.4	43.6	49.1
Death	Hospital-ICU-Post-ICU-Death	Apr	34.2	29.6	44.2	28.4	24.6	32.0	18.5	16.1	21.3	42.2	37.2	48.7
Death	Hospital-ICU-Post-ICU-Death	May	25.4	21.3	35.8	20.6	15.8	31.6	13.5	10.2	21.1	30.6	23.9	46.6
Death	Hospital-ICU-Post-ICU-Death	Jun	23.8	20.4	35.0	19.8	15.1	30.1	12.8	9.7	20.1	29.7	22.7	44.5
Discharge	Hospital-Discharge	Feb	34.0	31.5	36.1	22.4	20.9	23.9	7.6	7.2	8.5	48.0	44.9	52.4
Discharge	Hospital-Discharge	Mar	17.3	17.0	17.5	11.3	10.9	11.7	3.8	3.7	4.1	24.4	23.7	25.3
Discharge	Hospital-Discharge	Apr	16.5	16.1	16.9	10.8	10.5	11.2	3.7	3.6	3.9	23.7	22.8	24.4
Discharge	Hospital-Discharge	May	11.6	11.0	12.2	7.7	7.3	8.1	2.7	2.5	2.8	16.5	15.6	17.6
Discharge	Hospital-Discharge	Jun	7.7	6.9	8.7	5.1	4.7	5.7	1.8	1.6	2.0	10.9	10.2	12.2
Discharge	Hospital-ICU-Post-ICU-Discharge	Feb	44.7	42.6	48.3	39.1	36.2	43.8	27.1	24.8	29.9	55.6	51.3	62.0
Discharge	Hospital-ICU-Post-ICU-Discharge	Mar	47.0	45.9	48.7	42.4	40.7	43.0	29.0	27.6	29.6	59.9	57.4	61.1
Discharge	Hospital-ICU-Post-ICU-Discharge	Apr	40.1	37.6	42.6	35.3	33.8	37.8	24.1	23.1	26.0	50.4	47.7	53.7
Discharge	Hospital-ICU-Post-ICU-Discharge	May	27.5	24.5	32.7	24.4	20.3	28.7	16.5	13.9	19.6	34.5	28.7	40.3
Discharge	Hospital-ICU-Post-ICU-Discharge	Jun	25.9	22.3	30.8	22.9	19.5	27.8	15.7	13.5	19.0	32.6	27.5	39.3
Death	Hospital-Death	Feb	27.5	24.9	29.9	18.2	16.3	19.7	9.1	8.1	9.5	34.7	31.6	38.3
Death	Hospital-Death	Mar	8.4	8.2	8.6	5.5	5.3	5.7	2.7	2.5	2.8	10.7	10.4	11.1
Death	Hospital-Death	Apr	10.1	9.8	10.6	6.7	6.4	7.0	3.3	3.1	3.4	13.0	12.4	13.6
Death	Hospital-Death	May	9.7	8.3	11.0	6.4	5.7	7.5	3.1	2.7	3.6	12.4	11.0	14.7
Death	Hospital-Death	Jun	6.5	4.7	8.3	4.2	3.1	5.6	2.1	1.5	2.7	8.3	6.0	10.7
Death	Hospital-ICU-Death	Feb	20.9	19.1	23.0	16.0	14.5	17.9	9.6	8.5	10.5	26.1	23.3	29.5
Death	Hospital-ICU-Death	Mar	18.6	17.9	19.5	14.5	13.8	15.0	8.5	8.0	8.8	23.6	22.3	24.1
Death	Hospital-ICU-Death	Apr	19.6	17.4	21.6	15.4	13.9	16.4	9.0	8.1	9.6	25.0	22.5	26.5
Death	Hospital-ICU-Death	May	8.7	6.6	11.2	6.5	4.7	8.4	3.8	2.8	5.0	10.4	7.6	13.5
Death	Hospital-ICU-Death	Jun	7.1	5.7	9.2	5.5	3.7	7.9	3.3	2.2	4.6	9.0	6.0	12.7
Death	Averaged over pathways	Feb	26.6	25.0	29.1	17.8	17.1	19.9	8.9	8.5	10.3	33.7	31.4	36.4
Death	Averaged over pathways	Mar	10.5	10.2	10.8	6.7	6.3	6.9	3.2	2.9	3.3	13.2	12.7	13.9

Death	Averaged over pathways	Apr	11.3	10.9	11.8	7.1	6.9	7.7	3.2	3.2	3.7	14.5	13.8	15.5
Death	Averaged over pathways	May	10.1	8.9	11.9	6.5	5.9	7.4	3.0	2.9	3.6	12.4	11.3	14.4
Death	Averaged over pathways	Jun	7.2	5.5	9.0	4.7	3.3	6.0	2.3	1.5	2.8	9.1	6.6	11.7
Discharge	Averaged over pathways	Feb	34.9	33.1	36.7	24.6	22.8	26.1	8.8	7.9	9.6	49.5	46.3	52.3
Discharge	Averaged over pathways	Mar	19.5	19.4	19.7	12.7	12.2	13.5	4.3	4.1	4.7	27.7	27.1	29.0
Discharge	Averaged over pathways	Apr	17.6	17.3	18.0	11.6	11.2	12.2	4.0	3.8	4.3	25.3	24.2	26.2
Discharge	Averaged over pathways	May	12.0	11.5	12.6	7.8	7.4	8.7	2.9	2.5	3.0	17.1	16.3	18.4
Discharge	Averaged over pathways	Jun	8.1	7.2	9.1	5.1	4.8	5.8	1.7	1.7	2.0	11.3	10.6	12.6

Table A.15: Summaries of times from hospital admission to final events (total length of stay), by pathway through hospital and month of admission, conditional on experiencing that final event and assuming missing outcomes are censoring at 1 day after last observed event.

Estimated total lengths of stay in hospital by month of admission, averaged over pathways and final outcomes, are given in Table A.16, under the censoring assumption only:

**Month**	**Mean**	**95% CI of Mean**		**Median**	**95% CI of Median**		**25%-ile**	**95% CI of 25%-ile**		**75%-ile**	**95% CI of 75%-ile**	
Feb	32.0	30.7	33.6	21.2	20.5	22.8	8.9	8.2	9.5	43.1	41.6	45.9
Mar	16.8	16.6	17.1	10.3	9.8	10.5	3.7	3.6	3.9	22.9	22.4	24.0
Apr	16.2	15.8	16.5	10.3	10.1	10.7	3.8	3.7	4.0	22.7	22.2	23.5
May	11.7	11.1	12.4	7.7	7.2	8.1	2.8	2.6	3.0	16.7	15.7	17.4
Jun	8.0	7.2	8.8	5.2	4.7	5.8	1.8	1.6	2.1	11.5	10.2	12.7

Table A.16: Summaries of lengths of stay in hospital (total time in hospital), by month of admission and averaged over pathways and final outcomes, by month of admission, assuming missing outcomes are censoring at 1 day after last observed event.

A.3.3 Model regressed on hospital bed capacity

Parameter estimates from this model are given in Table A.17:

				Estimate		SE		OR or ETR		Lower		Upper	
**From**	**To**	**Distribution**	**Parameter**	**Censor**	**Missing**	**Censor**	**Missing**	**Censor**	**Missing**	**Censor**	**Missing**	**Censor**	**Missing**
Hospital	Discharge		$\pi_{1,2}$	0.6655	0.6658	0.0234	0.0234						
Hospital	ICU		$\pi_{1,3}$	0.1020	0.1018	0.0199	0.0199						
Hospital	Death		$\pi_{1,5}$	0.2325	0.2324	0.0143	0.0143						
Hospital	Discharge		$\pi_{1,2}$Med.	-0.1409	-0.1451	0.0534	0.0535	0.8686	0.8650	0.7823	0.7788	0.9644	0.9606
Hospital	Discharge		$\pi_{1,2}$Small	-0.7980	-0.7834	0.0598	0.0595	0.4502	0.4568	0.4004	0.4065	0.5062	0.5134
Hospital	ICU		$\pi_{1,3}$Med.	0.1103	0.1115	0.0351	0.0351	1.1166	1.1179	1.0425	1.0437	1.1961	1.1974
Hospital	ICU		$\pi_{1,3}$Small	-0.2574	-0.2565	0.0342	0.0342	0.7731	0.7737	0.7230	0.7236	0.8267	0.8274
Hospital	Discharge	gengamma	$\mu_{1,2}$	2.8221	2.8217	0.0131	0.0131						
Hospital	Discharge	gengamma	$\sigma_{1,2}$	1.0662	1.0666	0.0068	0.0068						
Hospital	Discharge	gengamma	$Q_{1,2}$	1.2751	1.2760	0.0185	0.0185						
Hospital	Discharge	gengamma	$T_{1,2}$Med.	0.1544	0.1566	0.0197	0.0197	1.1670	1.1695	1.1229	1.1252	1.2129	1.2155
Hospital	Discharge	gengamma	$T_{1,2}$Small	0.4556	0.4543	0.0172	0.0172	1.5771	1.5751	1.5247	1.5229	1.6312	1.6292
Hospital	ICU	gengamma	$\mu_{1,3}$	0.8398	0.8486	0.0432	0.0431						
Hospital	ICU	gengamma	$\sigma_{1,3}$	1.2415	1.2441	0.0124	0.0123						
Hospital	ICU	gengamma	$Q_{1,3}$	-0.2239	-0.2101	0.0603	0.0600						
Hospital	ICU	gengamma	$T_{1,3}$Med.	0.0158	0.0175	0.0624	0.0625	1.0159	1.0177	0.8990	0.9003	1.1481	1.1503
Hospital	ICU	gengamma	$T_{1,3}$Small	-0.0185	-0.0255	0.0717	0.0720	0.9817	0.9748	0.8530	0.8466	1.1297	1.1225
Hospital	Death	gengamma	$\mu_{1,5}$	1.8675	1.8685	0.0192	0.0192						
Hospital	Death	gengamma	$\sigma_{1,5}$	1.0470	1.0471	0.0078	0.0078						
Hospital	Death	gengamma	$Q_{1,5}$	0.3217	0.3247	0.0277	0.0278						
Hospital	Death	gengamma	$T_{1,5}$Med.	0.0646	0.0680	0.0312	0.0312	1.0667	1.0704	1.0034	1.0068	1.1340	1.1380
Hospital	Death	gengamma	$T_{1,5}$Small	0.1945	0.1964	0.0318	0.0318	1.2148	1.2170	1.1413	1.1434	1.2930	1.2954
ICU	Post-ICU		$\pi_{3,4}$	0.5548	0.5549	0.0373	0.0373						
ICU	Death		$\pi_{3,5}$	0.4452	0.4451	0.0373	0.0373						

ICU	Post-ICU		$\pi_{3,4}$Med.	0.1041	0.1042	0.1007	0.1007	1.1097	1.1098	0.9110	0.9111	1.3518	1.3519
ICU	Post-ICU		$\pi_{3,4}$Small	-1.2325	-1.2359	0.1435	0.1437	0.2916	0.2906	0.2201	0.2193	0.3863	0.3851
ICU	Post-ICU	gamma	shape	1.2790	1.2789	0.0276	0.0276						
ICU	Post-ICU	gamma	rate	0.0757	0.0757	0.0353	0.0353						
ICU	Post-ICU	gamma	$T_{3,4}$Med.	0.0018	0.0010	0.0608	0.0609	1.0018	1.0010	0.8892	0.8885	1.1286	1.1278
ICU	Post-ICU	gamma	$T_{3,4}$Small	0.0861	0.0869	0.0577	0.0577	1.0899	1.0908	0.9734	0.9742	1.2205	1.2214
ICU	Death	gamma	shape	1.4954	1.4945	0.0324	0.0324						
ICU	Death	gamma	rate	0.1207	0.1206	0.0396	0.0396						
ICU	Death	gamma	$T_{3,5}$Med.	0.1132	0.1138	0.0601	0.0602	1.1199	1.1205	0.9954	0.9959	1.2600	1.2607
ICU	Death	gamma	$T_{3,5}$Small	-0.0400	-0.0402	0.1047	0.1047	0.9608	0.9606	0.7825	0.7824	1.1796	1.1795
Post-ICU	Discharge		$\pi_{4,2}$	0.8973	0.8974	0.0892	0.0892						
Post-ICU	Death		$\pi_{4,5}$	0.1027	0.1026	0.0892	0.0892						
Post-ICU	Discharge		$\pi_{4,2}$Med.	-0.0295	-0.0396	0.2457	0.2466	0.9709	0.9611	0.5998	0.5927	1.5715	1.5585
Post-ICU	Discharge		$\pi_{4,2}$Small	-0.4192	-0.4110	0.2750	0.2742	0.6575	0.6630	0.3835	0.3873	1.1273	1.1348
Post-ICU	Discharge	gengamma	$\mu_{4,2}$	3.0501	3.0507	0.0284	0.0285						
Post-ICU	Discharge	gengamma	$\sigma_{4,2}$	0.6846	0.6847	0.0202	0.0203						
Post-ICU	Discharge	gengamma	$Q_{4,2}$	0.6873	0.6901	0.0549	0.0553						
Post-ICU	Discharge	gengamma	$T_{4,2}$Med.	0.1656	0.1661	0.0533	0.0533	1.1801	1.1807	1.0631	1.0635	1.3101	1.3107
Post-ICU	Discharge	gengamma	$T_{4,2}$Small	0.2204	0.2205	0.0513	0.0513	1.2465	1.2467	1.1273	1.1274	1.3784	1.3786
Post-ICU	Death	gengamma	$\mu_{4,5}$	2.0590	2.0846	0.1337	0.1342						
Post-ICU	Death	gengamma	$\sigma_{4,5}$	0.8253	0.8285	0.0580	0.0570						
Post-ICU	Death	gengamma	$Q_{4,5}$	-0.2999	-0.2467	0.2644	0.2650						
Post-ICU	Death	gengamma	$T_{4,5}$Med.	0.0547	0.0493	0.1930	0.1938	1.0563	1.0506	0.7236	0.7185	1.5418	1.5361
Post-ICU	Death	gengamma	$T_{4,5}$Small	-0.1179	-0.1366	0.2235	0.2244	0.8887	0.8723	0.5735	0.5619	1.3772	1.3543

Table A.17: parameter estimates for the model regressed on hospital bed capacity, by missing outcome assumption: (a) censoring at 1 day after last observed event; (b) ignoring missing outcomes. OR refers to odds ratios for the probabilities of each transition in

each hospital bed capacity relative to large hospitals. ETR refers to the expected time ratios for the times of each transition in each hospital bed capacity relative to large hospitals.

Comparison of non-parametric and parametric cumulative incidence curves, under both the missing outcome assumptions, are shown in Figure A.5:

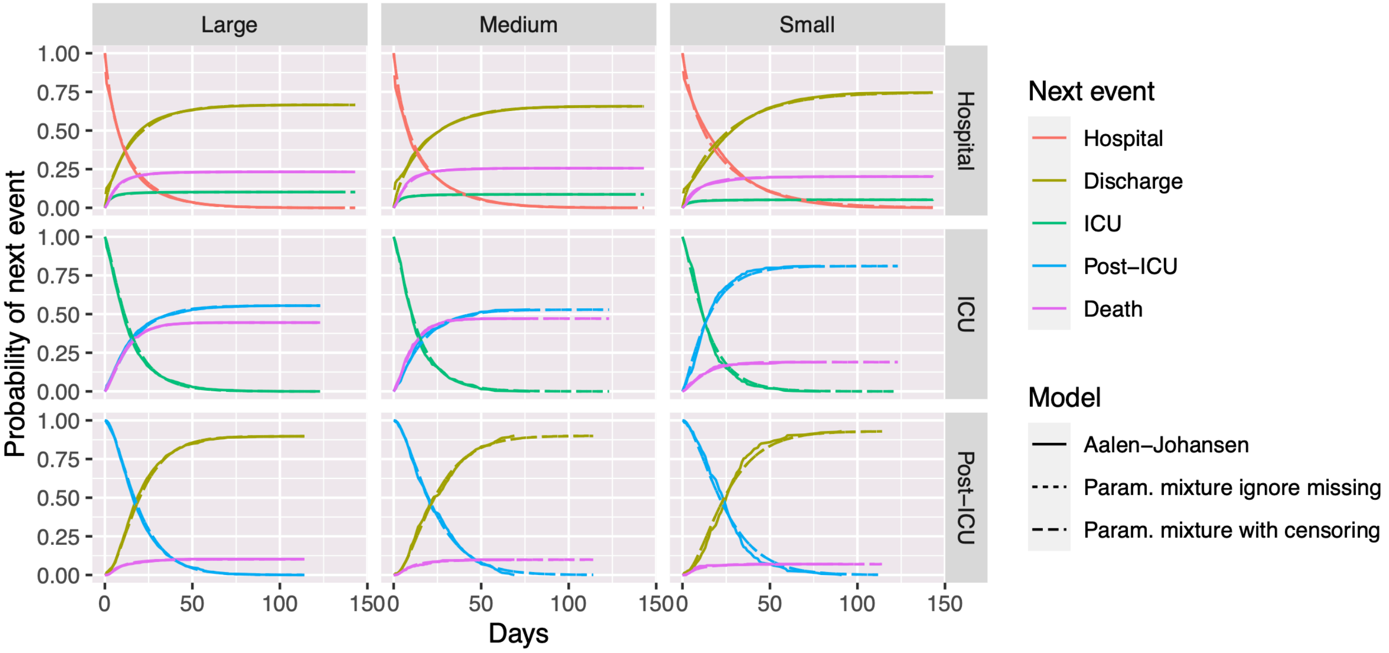

Figure A.5: Parametric versus non-parametric cumulative incidence estimates, by starting state (rows), next event (colours), hospital bed capacity (columns) and model (line type).

Estimated probabilities of next events are given in Table A.18:

			CENSORING			MISSING		
**From**	**Next Event**	**Hospital Bed Capacity**	**Pr(Next Event)**	**Lower**	**Upper**	**Pr(Next Event)**	**Lower**	**Upper**
Hospital	Discharge	Large	0.666	0.660	0.670	0.666	0.661	0.670
Hospital	Discharge	Medium	0.656	0.645	0.670	0.657	0.645	0.671
Hospital	Discharge	Small	0.747	0.737	0.760	0.746	0.734	0.755
Hospital	ICU	Large	0.102	0.100	0.105	0.102	0.098	0.105

Hospital	ICU	Medium	0.087	0.079	0.094	0.087	0.082	0.095
Hospital	ICU	Small	0.052	0.047	0.056	0.052	0.048	0.058
Hospital	Death	Large	0.233	0.226	0.238	0.232	0.228	0.237
Hospital	Death	Medium	0.256	0.245	0.266	0.256	0.242	0.266
Hospital	Death	Small	0.202	0.191	0.213	0.202	0.193	0.209
ICU	Post-ICU	Large	0.555	0.542	0.572	0.555	0.538	0.574
ICU	Post-ICU	Medium	0.529	0.495	0.568	0.529	0.487	0.572
ICU	Post-ICU	Small	0.810	0.774	0.841	0.811	0.773	0.850
ICU	Death	Large	0.445	0.428	0.458	0.445	0.426	0.462
ICU	Death	Medium	0.471	0.432	0.505	0.471	0.428	0.513
ICU	Death	Small	0.190	0.159	0.226	0.189	0.150	0.227
Post-ICU	Discharge	Large	0.897	0.883	0.911	0.897	0.883	0.910
Post-ICU	Discharge	Medium	0.900	0.852	0.924	0.901	0.869	0.932
Post-ICU	Discharge	Small	0.930	0.895	0.952	0.930	0.888	0.960
Post-ICU	Death	Large	0.103	0.089	0.117	0.103	0.090	0.117
Post-ICU	Death	Medium	0.100	0.076	0.148	0.099	0.068	0.131
Post-ICU	Death	Small	0.070	0.048	0.105	0.070	0.040	0.112

Table A.18: Estimated probabilities (95% confidence intervals) of next events, given current state, by hospital bed capacity and missing outcome assumption: (a) censoring at 1 day after last observed event; (b) ignoring missing outcomes.

Estimated probabilities of final events are given in Table A.19:

		**CENSORING**			**MISSING**		
**Final Event**	**Hospital Bed Capacity**	**Pr(Final Event)**	**Lower**	**Upper**	**Pr(Final Event)**	**Lower**	**Upper**
Death	Large	0.284	0.279	0.289	0.283	0.277	0.288
Death	Medium	0.302	0.290	0.315	0.302	0.292	0.313
Death	Small	0.214	0.206	0.223	0.214	0.205	0.223

Discharge	Large	0.716	0.711	0.721	0.717	0.712	0.723
Discharge	Medium	0.698	0.685	0.710	0.698	0.687	0.708
Discharge	Small	0.786	0.777	0.794	0.786	0.777	0.795

Table A.19: Estimated probabilities (95% confidence intervals) of final events, given current state (hospital-fatality risks and complement), by hospital bed capacity and missing outcome assumption: (a) censoring at 1 day after last observed event; (b) ignoring missing outcomes.

Estimated times to next events, under the censoring assumption only, are given in Table A.20:

From	Next Event	Hospital Bed Capacity	Mean	95% CI of Mean		Median	95% CI of Median		25%-ile	95% CI of 25%-ile		75%-ile	95% CI of 75%-ile	
Hospital	Discharge	Large	15.4	15.2	15.6	10.0	9.8	10.1	3.5	3.4	3.5	21.7	21.3	22.0
	Discharge	Medium	17.9	17.4	18.5	11.6	11.2	12.1	4.0	3.9	4.2	25.4	24.3	26.3
	Discharge	Small	24.2	23.3	24.8	15.7	15.2	16.1	5.5	5.3	5.6	34.3	33.1	35.1
	ICU	Large	6.4	5.8	6.9	2.5	2.4	2.7	1.1	1.1	1.2	6.0	5.7	6.3
	ICU	Medium	6.5	5.8	7.7	2.6	2.3	2.8	1.1	1.0	1.2	6.1	5.5	6.8
	ICU	Small	6.3	5.4	7.1	2.5	2.3	2.7	1.1	1.0	1.2	5.9	5.4	6.5
	Death	Large	9.2	8.9	9.5	5.8	5.7	5.9	2.7	2.7	2.8	11.5	11.4	11.8
	Death	Medium	9.8	9.2	10.1	6.2	5.8	6.5	2.9	2.8	3.1	12.3	11.6	13.0
	Death	Small	11.1	10.4	11.6	7.0	6.7	7.4	3.3	3.2	3.5	14.0	13.4	14.8
ICU	Post-ICU	Large	16.9	16.2	17.4	12.8	12.2	13.5	6.1	5.7	6.5	23.3	22.2	24.6
	Post-ICU	Medium	16.9	15.4	19.1	12.7	11.4	14.3	6.1	5.4	6.7	23.3	20.9	26.3
	Post-ICU	Small	15.5	13.5	16.7	11.7	10.6	12.9	5.6	5.0	6.2	21.4	19.4	23.4
	Death	Large	12.4	11.8	12.9	9.8	9.4	10.2	5.0	4.8	5.3	17.0	16.3	17.7
	Death	Medium	11.1	10.2	12.3	8.7	7.8	9.9	4.5	4.0	5.1	15.2	13.5	17.1
	Death	Small	12.9	10.5	15.2	10.2	8.6	12.1	5.2	4.4	6.2	17.7	15.1	20.9

Post-ICU	Discharge	Large	21.1	20.4	21.8	17.9	17.3	18.5	10.5	10.0	10.8	28.3	27.4	29.2
	Discharge	Medium	24.9	22.6	26.7	21.1	19.4	22.8	12.4	11.4	13.4	33.4	30.3	36.2
	Discharge	Small	26.3	23.6	28.3	22.3	20.6	24.2	13.1	11.9	14.2	35.3	32.6	38.6
	Death	Large	13.1	11.4	18.7	8.5	7.7	9.8	4.9	4.4	5.7	15.3	13.7	17.9
	Death	Medium	13.9	10.9	23.0	9.0	6.5	14.1	5.2	3.7	8.2	16.1	11.4	24.1
	Death	Small	11.7	7.7	21.3	7.6	5.7	11.8	4.4	3.2	6.8	13.6	10.2	20.4

Table A.20: Summaries of times from current state to next event, conditional on experiencing that next event, by hospital bed capacity, assuming missing outcomes are censoring at 1 day after last observed event.

Estimated times to final events (total length of stay in hospital), by pathway through hospital, are given in Table A.21, under the censoring assumption only:

**Outcome**	**Pathway**	**Hospital Bed Capacity**	**Mean**	**95% CI of Mean**		**Median**	**95% CI of Median**		**25%-ile**	**95% CI of 25%-ile**		**75%-ile**	**95% CI of 75%-ile**	
Death	Hospital-Death	Large	9.2	8.9	9.4	5.8	5.6	6.0	2.8	2.6	2.8	11.3	11.2	11.9
Death	Hospital-Death	Medium	9.8	9.3	10.3	6.0	5.9	6.5	2.9	2.8	3.2	12.0	11.8	13.1
Death	Hospital-Death	Small	11.1	10.7	11.6	6.8	6.6	7.5	3.3	3.2	3.5	14.2	13.1	15.0
Death	Hospital-ICU-Death	Large	18.8	18.2	19.6	14.7	14.2	15.2	8.5	8.2	8.9	23.4	22.8	24.5
Death	Hospital-ICU-Death	Medium	17.6	16.3	19.4	13.4	12.6	14.7	7.9	7.5	8.6	21.8	20.7	24.0
Death	Hospital-ICU-Death	Small	19.2	16.9	23.3	15.1	13.3	17.3	8.8	7.7	10.1	24.4	21.2	28.4
Death	Hospital-ICU-Post-ICU-Death	Large	36.5	35.0	42.7	30.4	28.8	31.5	19.6	18.5	20.6	45.4	43.6	48.5
Death	Hospital-ICU-Post-ICU-Death	Medium	37.3	32.5	46.7	31.0	26.4	36.6	20.5	17.1	24.0	47.2	40.1	54.7
Death	Hospital-ICU-Post-ICU-Death	Small	33.5	30.1	44.3	27.7	25.4	32.6	17.8	16.4	21.2	41.4	38.5	50.0
Discharge	Hospital-Discharge	Large	15.4	15.1	15.6	10.1	9.6	10.3	3.5	3.3	3.7	21.8	21.1	22.3
Discharge	Hospital-Discharge	Medium	17.9	17.3	18.4	11.9	11.2	12.2	4.1	3.8	4.3	25.7	24.7	26.7
Discharge	Hospital-Discharge	Small	24.2	23.6	24.9	15.5	15.1	16.5	5.6	5.1	5.9	34.5	33.0	35.8

Discharge	Hospital-ICU-Post-ICU-Discharge	Large	44.4	43.0	45.4	39.4	38.5	40.3	27.1	26.3	27.8	55.8	54.3	57.1
Discharge	Hospital-ICU-Post-ICU-Discharge	Medium	48.3	46.1	51.1	43.3	40.2	45.9	29.8	27.5	31.5	61.4	56.7	64.9
Discharge	Hospital-ICU-Post-ICU-Discharge	Small	48.1	45.5	51.2	43.0	40.7	45.6	29.5	27.9	31.4	60.8	57.3	63.7
Death	Averaged over pathways	Large	11.3	11.2	11.6	7.0	6.6	7.3	3.3	3.0	3.4	14.5	13.5	15.2
Death	Averaged over pathways	Medium	11.3	10.8	11.9	7.5	6.7	7.6	3.4	3.1	3.5	14.8	13.3	15.5
Death	Averaged over pathways	Small	11.8	11.2	12.4	7.6	6.8	8.0	3.3	3.2	3.8	15.3	13.9	16.0
Discharge	Averaged over pathways	Large	17.4	17.2	17.7	11.1	10.8	11.7	3.8	3.6	4.1	24.9	24.0	25.4
Discharge	Averaged over pathways	Medium	19.7	19.1	20.5	13.0	12.3	13.3	4.5	4.1	4.7	28.5	27.4	29.2
Discharge	Averaged over pathways	Small	25.4	24.9	26.2	16.6	16.0	17.8	5.7	5.4	6.3	36.6	34.6	38.0

Table A.21 Summaries of times from hospital admission to final events (total length of stay), by hospital bed capacity and pathway through hospital, conditional on experiencing that final event and assuming missing outcomes are censoring at 1 day after last observed event.

Estimated total lengths of stay in hospital, by hospital bed capacity and averaged over pathways and final outcomes, are given in Table A.22, under the censoring assumption only:

**Hospital Bed Capacity**	**Mean**	**95% CI of Mean**		**Median**	**95% CI of Median**		**25%-ile**	**95% CI of 25%-ile**		**75%-ile**	**95% CI of 75%-ile**	
Large	15.7	15.5	15.9	9.7	9.4	9.9	3.6	3.5	3.7	21.3	21.1	22.1
Medium	17.2	16.8	17.7	10.4	10.1	10.7	3.8	3.7	4.1	23.2	22.8	24.5
Small	22.5	21.8	23.0	13.7	13.2	14.2	4.9	4.8	5.2	32.0	30.7	32.6

Table A.22: Summaries of lengths of stay in hospital (total time in hospital), by hospital bed capacity and averaged over pathways and final outcomes, assuming missing outcomes are censoring at 1 day after last observed event.

A.3.4 Model regressed on both month of admission and hospital bed capacity

The effect of hospital bed capacity on probabilities of next events from an admitting hospital ward and from ICU remains significant once adjusted for month of admission, while its effect on transitions from post-ICU remains non-significant. The predicted

probabilities by hospital bed capacity and month of admission are shown in Figure A.6: probabilities of severe events (ICU admission, death) decrease with calendar month and are smaller in the smallest hospitals than in others; while the corresponding probabilities of discharge and transfer to a post-ICU ward increase with time and are largest in the smallest hospitals. Note that the absolute differences between the probabilities by hospital size also decrease with calendar month.

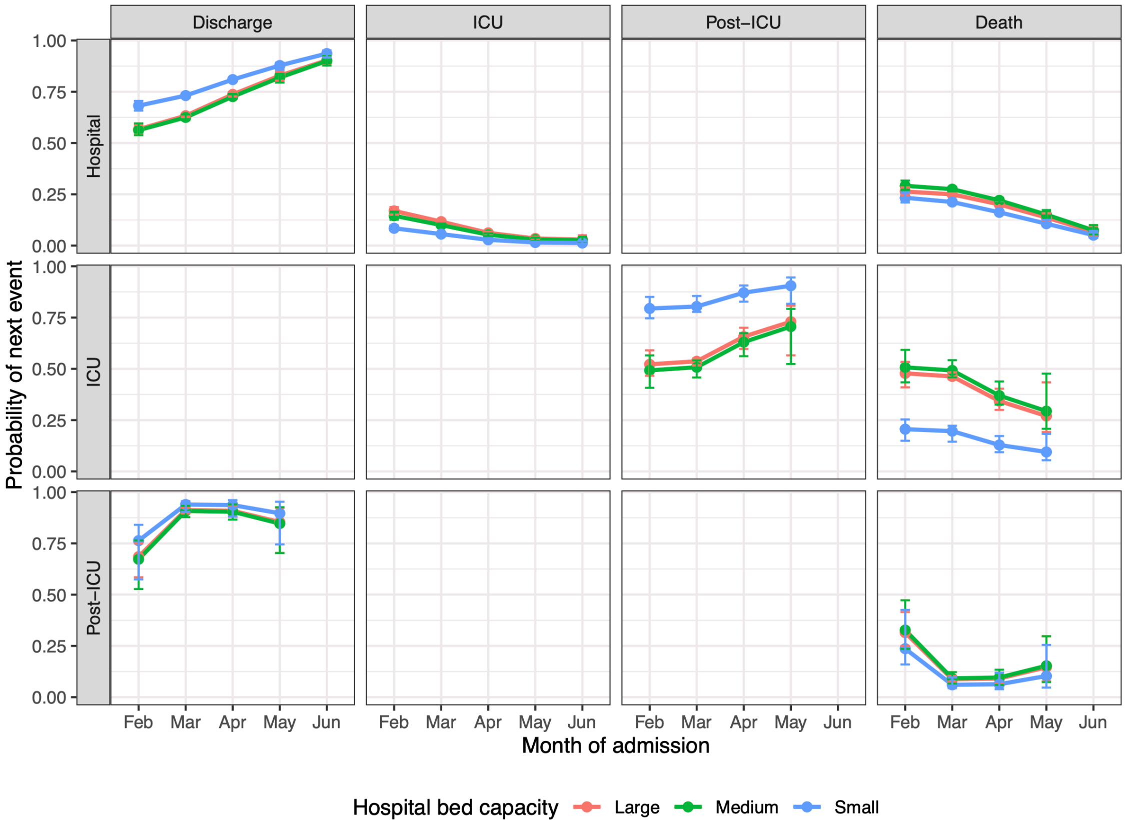

Figure A.6: Predicted probabilities of moving to the next event, by current state (rows), next event (columns), month of admission (x-axis) and hospital bed capacity (colours).

The state-specific fatality risks, weighted by the probabilities of each pathway through hospital, result in a hospital-fatality risk that decreases with calendar month and is smallest in the smallest hospitals (Figure A.7).

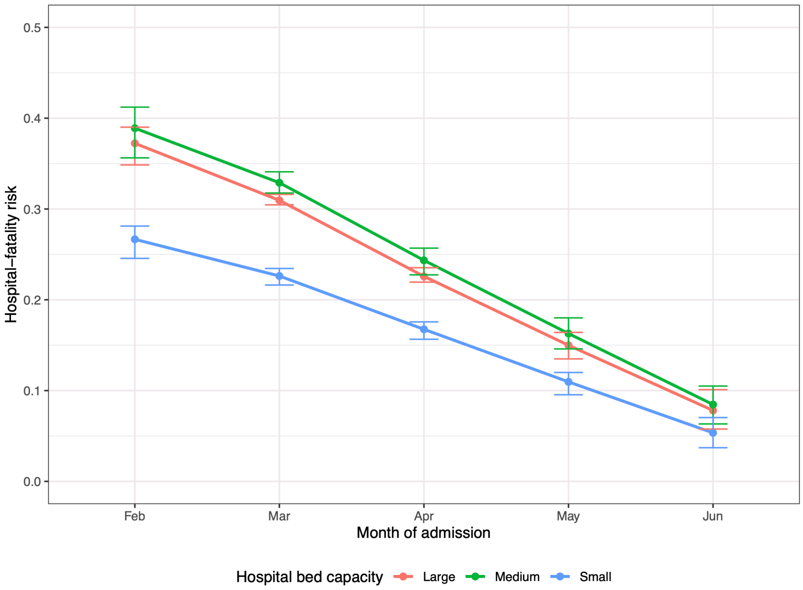

Figure A.7: Hospital-fatality risk, averaged over all pathways through hospital, by month of admission (x-axis) and hospital bed capacity (colours).

Adjusted for month of admission, the effect of hospital bed capacity is similar to the unadjusted effect: LoS among survivors decreases with increasing bed capacity, whereas there is little effect on times to death, regardless of which hospital ward the patient is in (Figure A.8). Month of admission has significant effects, with LoS in each stage of hospital decreasing over time for survivors (Figure A.8).

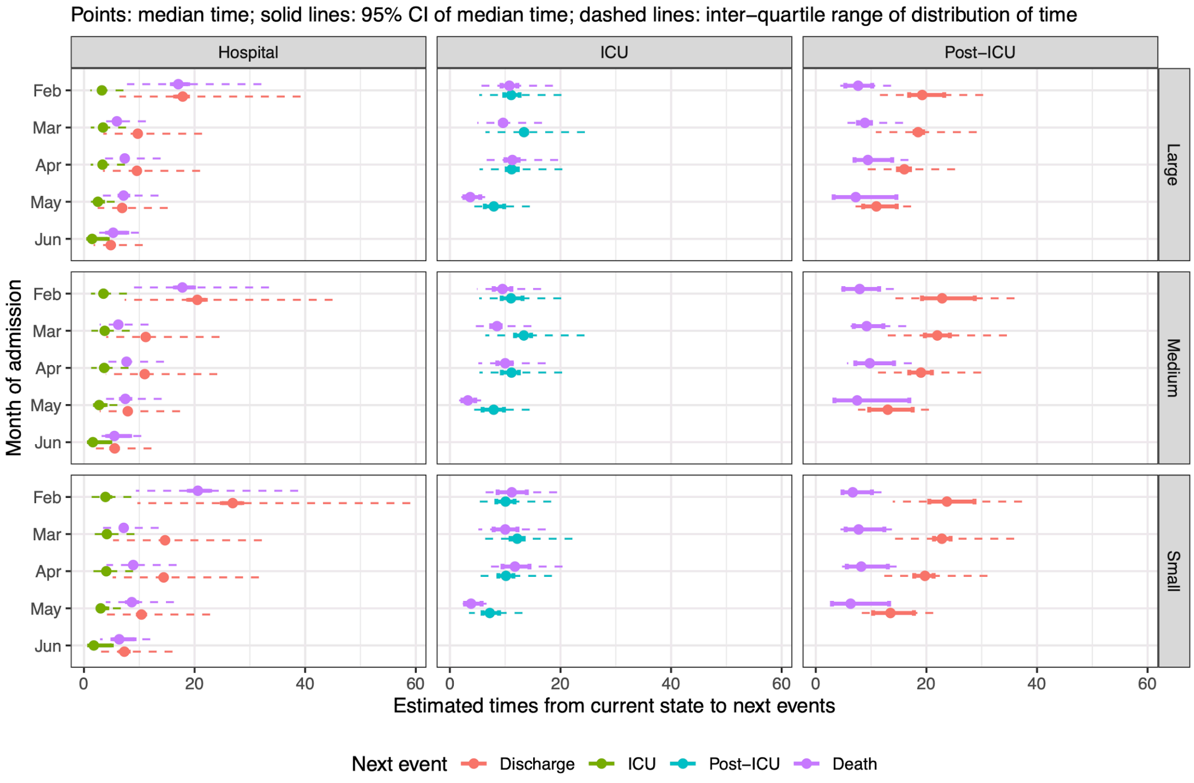

Figure A.8: Summaries of distributions of lengths of stay in hospital, by current state (columns), next event (colours), month of admission (y-axis) and hospital bed capacity (rows). The 95% CI of the median times (solid lines) represent uncertainty in the estimate, whereas the inter-quartile range of the distribution (dashed lines) represents heterogeneity in the population.

Parameter estimates from this model are given in Table A.23:

				Estimate		SE		OR or ETR		Lower		Upper	
**From**	**To**	**Distribution**	**Parameter**	**Censor**	**Missing**	**Censor**	**Missing**	**Censor**	**Missing**	**Censor**	**Missing**	**Censor**	**Missing**
Hospital	Discharge		$\pi_{1,2}$	0.6332	0.6330	0.0261	0.0260						
Hospital	ICU		$\pi_{1,3}$	0.1168	0.1170	0.0221	0.0221						
Hospital	Death		$\pi_{1,5}$	0.2499	0.2501	0.0162	0.0162						
Hospital	Discharge		$\pi_{1,2}$Feb	0.4808	0.4628	0.0767	0.0770	1.6174	1.5885	1.3916	1.3659	1.8798	1.8474
Hospital	Discharge		$\pi_{1,2}$Apr	-0.7842	-0.7873	0.0509	0.0510	0.4565	0.4551	0.4131	0.4118	0.5044	0.5029
Hospital	Discharge		$\pi_{1,2}$May	-1.4867	-1.5563	0.1476	0.1523	0.2261	0.2109	0.1693	0.1565	0.3020	0.2843

Hospital	Discharge		$\pi_{1,2}$Jun	-1.7283	-1.7360	0.2802	0.2828	0.1776	0.1762	0.1025	0.1012	0.3075	0.3067
Hospital	Discharge		$\pi_{1,2}$Med.	-0.1422	-0.1426	0.0538	0.0538	0.8675	0.8671	0.7806	0.7803	0.9640	0.9636
Hospital	Discharge		$\pi_{1,2}$Small	-0.8785	-0.8781	0.0599	0.0599	0.4154	0.4156	0.3694	0.3695	0.4672	0.4674
Hospital	ICU		$\pi_{1,3}$Feb	0.1636	0.1566	0.0608	0.0608	1.1777	1.1695	1.0454	1.0381	1.3267	1.3176
Hospital	ICU		$\pi_{1,3}$Apr	-0.3707	-0.3728	0.0305	0.0306	0.6903	0.6888	0.6502	0.6487	0.7328	0.7313
Hospital	ICU		$\pi_{1,3}$May	-0.8710	-0.8699	0.0768	0.0766	0.4185	0.4190	0.3601	0.3606	0.4865	0.4869
Hospital	ICU		$\pi_{1,3}$Jun	-1.6725	-1.6956	0.1882	0.1895	0.1878	0.1835	0.1299	0.1266	0.2715	0.2660
Hospital	ICU		$\pi_{1,3}$Med.	0.1088	0.1071	0.0352	0.0353	1.1150	1.1131	1.0405	1.0388	1.1947	1.1927
Hospital	ICU		$\pi_{1,3}$Small	-0.3066	-0.3099	0.0344	0.0344	0.7359	0.7335	0.6879	0.6856	0.7873	0.7847
Hospital	Discharge	gamma	shape	0.8093	0.8081	0.0074	0.0074						
Hospital	Discharge	gamma	rate	0.0524	0.0522	0.0120	0.0120						
Hospital	Discharge	gamma	$T_{1,2}$Feb	-0.6069	-0.6059	0.0370	0.0370	0.5451	0.5456	0.5070	0.5074	0.5860	0.5866
Hospital	Discharge	gamma	$T_{1,2}$Apr	0.0167	0.0193	0.0161	0.0161	1.0168	1.0195	0.9853	0.9879	1.0494	1.0522
Hospital	Discharge	gamma	$T_{1,2}$May	0.3434	0.3552	0.0325	0.0324	1.4097	1.4265	1.3227	1.3387	1.5025	1.5200
Hospital	Discharge	gamma	$T_{1,2}$Jun	0.6973	0.7179	0.0556	0.0553	2.0083	2.0501	1.8010	1.8394	2.2394	2.2850
Hospital	Discharge	gamma	$T_{1,2}$Med.	-0.1377	-0.1362	0.0205	0.0205	0.8713	0.8727	0.8369	0.8382	0.9071	0.9085
Hospital	Discharge	gamma	$T_{1,2}$Small	-0.4097	-0.4088	0.0180	0.0180	0.6638	0.6644	0.6408	0.6414	0.6877	0.6884
Hospital	ICU	gamma	shape	0.8011	0.8031	0.0201	0.0200						
Hospital	ICU	gamma	rate	0.1454	0.1455	0.0304	0.0304						
Hospital	ICU	gamma	$T_{1,3}$Feb	0.0635	0.0648	0.0769	0.0769	1.0656	1.0669	0.9164	0.9177	1.2390	1.2404
Hospital	ICU	gamma	$T_{1,3}$Apr	0.0268	0.0273	0.0544	0.0544	1.0272	1.0277	0.9232	0.9237	1.1429	1.1434
Hospital	ICU	gamma	$T_{1,3}$May	0.3148	0.3265	0.1669	0.1660	1.3700	1.3861	0.9877	1.0011	1.9002	1.9192
Hospital	ICU	gamma	$T_{1,3}$Jun	0.8443	1.0276	0.4786	0.4445	2.3263	2.7944	0.9105	1.1692	5.9439	6.6786
Hospital	ICU	gamma	$T_{1,3}$Med.	-0.0835	-0.0837	0.0563	0.0563	0.9199	0.9197	0.8238	0.8237	1.0272	1.0270
Hospital	ICU	gamma	$T_{1,3}$Small	-0.1811	-0.1794	0.0642	0.0641	0.8344	0.8358	0.7358	0.7371	0.9462	0.9477
Hospital	Death	gamma	shape	1.1773	1.1770	0.0131	0.0131						

Hospital	Death	gamma	rate	0.1455	0.1456	0.0182	0.0182						
Hospital	Death	gamma	$T_{1,5}$Feb	-1.0534	-1.0640	0.0469	0.0471	0.3487	0.3451	0.3181	0.3146	0.3823	0.3785
Hospital	Death	gamma	$T_{1,5}$Apr	-0.2149	-0.2161	0.0248	0.0248	0.8066	0.8056	0.7684	0.7675	0.8467	0.8457
Hospital	Death	gamma	$T_{1,5}$May	-0.1857	-0.1847	0.0654	0.0653	0.8305	0.8313	0.7306	0.7314	0.9441	0.9449
Hospital	Death	gamma	$T_{1,5}$Jun	0.1192	0.1266	0.1688	0.1686	1.1266	1.1350	0.8092	0.8155	1.5686	1.5796
Hospital	Death	gamma	$T_{1,5}$Med.	-0.0421	-0.0425	0.0275	0.0275	0.9588	0.9584	0.9085	0.9081	1.0119	1.0115
Hospital	Death	gamma	$T_{1,5}$Small	-0.1884	-0.1885	0.0280	0.0280	0.8283	0.8282	0.7841	0.7840	0.8750	0.8750
ICU	Post-ICU		$\pi_{3,4}$	0.5369	0.5369	0.0413	0.0413						
ICU	Death		$\pi_{3,5}$	0.4631	0.4631	0.0413	0.0413						
ICU	Post-ICU		$\pi_{3,4}$Feb	0.0598	0.0602	0.1396	0.1396	1.0616	1.0620	0.8075	0.8078	1.3957	1.3963
ICU	Post-ICU		$\pi_{3,4}$Apr	-0.5024	-0.5019	0.1045	0.1044	0.6051	0.6054	0.4931	0.4933	0.7425	0.7429
ICU	Post-ICU		$\pi_{3,4}$MayJun	-0.8463	-0.8384	0.3006	0.3001	0.4290	0.4324	0.2380	0.2401	0.7733	0.7786
ICU	Post-ICU		$\pi_{3,4}$Med.	0.1170	0.1167	0.1015	0.1015	1.1241	1.1238	0.9212	0.9210	1.3717	1.3713
ICU	Post-ICU		$\pi_{3,4}$Small	-1.2624	-1.2634	0.1445	0.1445	0.2830	0.2827	0.2132	0.2130	0.3756	0.3753
ICU	Post-ICU	gamma	shape	1.2917	1.2914	0.0276	0.0276						
ICU	Post-ICU	gamma	rate	0.0729	0.0729	0.0368	0.0368						
ICU	Post-ICU	gamma	$T_{3,4}$Feb	0.1906	0.1895	0.0822	0.0822	1.2100	1.2086	1.0299	1.0287	1.4215	1.4200
ICU	Post-ICU	gamma	$T_{3,4}$Apr	0.1827	0.1821	0.0533	0.0534	1.2004	1.1997	1.0812	1.0806	1.3327	1.3320
ICU	Post-ICU	gamma	$T_{3,4}$May	0.5237	0.5251	0.1357	0.1356	1.6882	1.6906	1.2939	1.2960	2.2027	2.2055
ICU	Post-ICU	gamma	$T_{3,4}$Med.	0.0025	0.0025	0.0609	0.0609	1.0025	1.0025	0.8898	0.8898	1.1294	1.1295
ICU	Post-ICU	gamma	$T_{3,4}$Small	0.0965	0.0962	0.0574	0.0574	1.1013	1.1010	0.9841	0.9837	1.2326	1.2322
ICU	Death	gamma	shape	1.5139	1.5122	0.0325	0.0325						
ICU	Death	gamma	rate	0.1244	0.1243	0.0408	0.0408						
ICU	Death	gamma	$T_{3,5}$Feb	-0.1119	-0.1112	0.0828	0.0828	0.8942	0.8948	0.7602	0.7607	1.0518	1.0526
ICU	Death	gamma	$T_{3,5}$Apr	-0.1611	-0.1613	0.0682	0.0683	0.8512	0.8510	0.7447	0.7445	0.9729	0.9729
ICU	Death	gamma	$T_{3,5}$May	0.9610	0.9612	0.2112	0.2113	2.6143	2.6149	1.7283	1.7282	3.9547	3.9564

ICU	Death	gamma	$T_{3,5}$Med.	0.1220	0.1212	0.0601	0.0601	1.1298	1.1289	1.0043	1.0033	1.2710	1.2701
ICU	Death	gamma	$T_{3,5}$Small	-0.0397	-0.0397	0.1042	0.1043	0.9611	0.9610	0.7835	0.7834	1.1789	1.1790
Post-ICU	Discharge		$\pi_{4,2}$	0.9126	0.9127	0.1062	0.1062						
Post-ICU	Death		$\pi_{4,5}$	0.0874	0.0873	0.1062	0.1062						
Post-ICU	Discharge		$\pi_{4,2}$Feb	1.5695	1.5595	0.2405	0.2409	4.8044	4.7564	2.9983	2.9666	7.6982	7.6261
Post-ICU	Discharge		$\pi_{4,2}$Apr	0.0461	0.0414	0.2307	0.2310	1.0472	1.0423	0.6663	0.6628	1.6460	1.6391
Post-ICU	Discharge		$\pi_{4,2}$MayJun	0.5809	0.6033	0.5038	0.4999	1.7877	1.8281	0.6659	0.6862	4.7992	4.8701
Post-ICU	Discharge		$\pi_{4,2}$Med.	0.0568	0.0593	0.2496	0.2495	1.0585	1.0611	0.6489	0.6507	1.7264	1.7303
Post-ICU	Discharge		$\pi_{4,2}$Small	-0.3945	-0.3839	0.2781	0.2772	0.6740	0.6812	0.3908	0.3957	1.1624	1.1727
Post-ICU	Discharge	gengamma	$\mu_{4,2}$	3.0839	3.0851	0.0298	0.0298						
Post-ICU	Discharge	gengamma	$\sigma_{4,2}$	0.6779	0.6779	0.0204	0.0204						
Post-ICU	Discharge	gengamma	$Q_{4,2}$	0.7002	0.7042	0.0557	0.0561						
Post-ICU	Discharge	gengamma	$T_{4,2}$Feb	0.0391	0.0396	0.0846	0.0846	1.0399	1.0404	0.8810	0.8814	1.2275	1.2281
Post-ICU	Discharge	gengamma	$T_{4,2}$Apr	-0.1444	-0.1441	0.0465	0.0465	0.8655	0.8658	0.7901	0.7903	0.9481	0.9484
Post-ICU	Discharge	gengamma	$T_{4,2}$MayJun	-0.5242	-0.5239	0.1295	0.1296	0.5920	0.5922	0.4593	0.4594	0.7631	0.7635
Post-ICU	Discharge	gengamma	$T_{4,2}$Med.	0.1718	0.1715	0.0530	0.0530	1.1874	1.1870	1.0703	1.0699	1.3174	1.3169
Post-ICU	Discharge	gengamma	$T_{4,2}$Small	0.2091	0.2095	0.0509	0.0509	1.2325	1.2331	1.1155	1.1160	1.3618	1.3625
Post-ICU	Death	gengamma	$\mu_{4,5}$	2.1252	2.1163	0.1542	0.1549						
Post-ICU	Death	gengamma	$\sigma_{4,5}$	0.8284	0.8279	0.0559	0.0565						
Post-ICU	Death	gengamma	$Q_{4,5}$	-0.2068	-0.2275	0.2716	0.2736						
Post-ICU	Death	gengamma	$T_{4,5}$Feb	-0.1477	-0.1412	0.1737	0.1736	0.8627	0.8683	0.6138	0.6178	1.2126	1.2203
Post-ICU	Death	gengamma	$T_{4,5}$Apr	0.0600	0.0539	0.1854	0.1855	1.0619	1.0554	0.7383	0.7336	1.5271	1.5183
Post-ICU	Death	gengamma	$T_{4,5}$MayJun	-0.2064	-0.2059	0.3882	0.3886	0.8135	0.8139	0.3802	0.3800	1.7410	1.7433
Post-ICU	Death	gengamma	$T_{4,5}$Med.	0.0355	0.0299	0.1948	0.1948	1.0361	1.0303	0.7073	0.7034	1.5178	1.5093
Post-ICU	Death	gengamma	$T_{4,5}$Small	-0.1366	-0.1249	0.2244	0.2242	0.8723	0.8826	0.5619	0.5687	1.3541	1.3696

Table A.23: parameter estimates for the model regressed on month of admission and hospital bed capacity, by missing outcome assumption: (a) censoring at 1 day after last observed event; (b) ignoring missing outcomes. OR refers to odds ratios for the

probabilities of each transition in each month relative to March and in each hospital bed capacity relative to large hospitals. ETR refers to the expected time ratios for the times of each transition in each month relative to March and in each hospital bed capacity relative to large hospitals.

Comparison of non-parametric and parametric cumulative incidence curves, under both the missing outcome assumptions, are shown in Figure A.9:

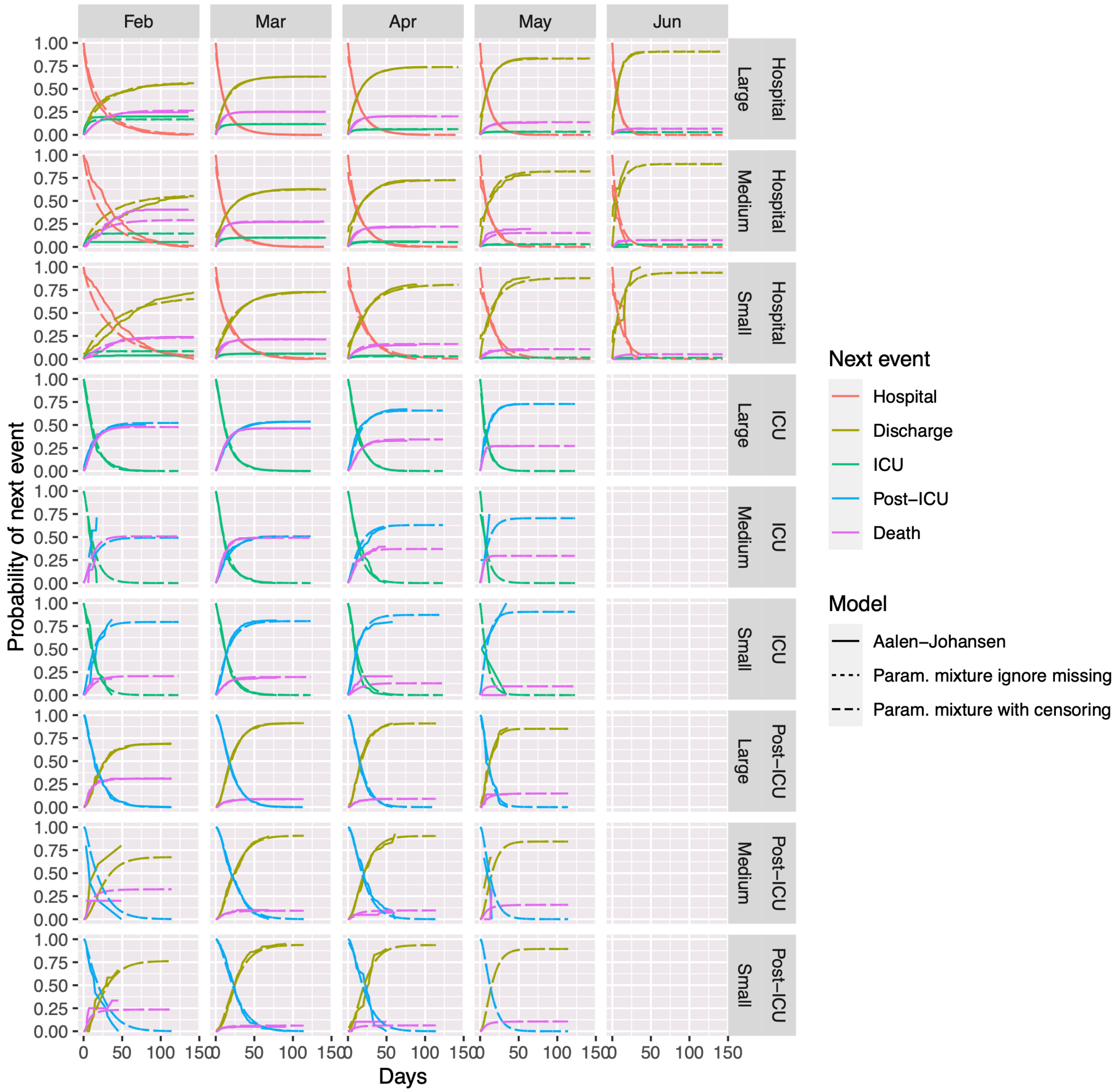

Figure A.9: Parametric versus non-parametric cumulative incidence estimates, by starting state (rows), next event (colours), month (columns), hospital bed capacity (rows) and model (line type). Note that for the ICU and post-ICU states, due to small sample sizes, May and June are combined and shown in the May column.

Estimated probabilities of next events are given in Table A.24:

From	Next Event	Hospital Bed Capacity	Month	CENSORING			MISSING		
				Pr(Next Event)	Lower	Upper	Pr(Next Event)	Lower	Upper
Hospital	Discharge	Large	Feb	0.567	0.551	0.584	0.570	0.537	0.590
Hospital	Discharge	Large	Mar	0.633	0.626	0.638	0.633	0.628	0.640
Hospital	Discharge	Large	Apr	0.737	0.728	0.746	0.737	0.728	0.745
Hospital	Discharge	Large	May	0.829	0.802	0.847	0.830	0.809	0.852
Hospital	Discharge	Large	Jun	0.903	0.874	0.930	0.905	0.872	0.923
Hospital	Discharge	Medium	Feb	0.563	0.542	0.586	0.565	0.537	0.588
Hospital	Discharge	Medium	Mar	0.625	0.606	0.638	0.625	0.612	0.638
Hospital	Discharge	Medium	Apr	0.726	0.709	0.741	0.727	0.711	0.742
Hospital	Discharge	Medium	May	0.819	0.792	0.839	0.821	0.796	0.842
Hospital	Discharge	Medium	Jun	0.900	0.871	0.929	0.902	0.869	0.922
Hospital	Discharge	Small	Feb	0.682	0.664	0.701	0.685	0.653	0.707
Hospital	Discharge	Small	Mar	0.731	0.723	0.742	0.732	0.721	0.740
Hospital	Discharge	Small	Apr	0.809	0.801	0.819	0.810	0.798	0.817
Hospital	Discharge	Small	May	0.878	0.859	0.892	0.879	0.861	0.894
Hospital	Discharge	Small	Jun	0.936	0.915	0.957	0.937	0.911	0.952
Hospital	ICU	Large	Feb	0.169	0.150	0.185	0.167	0.145	0.184
Hospital	ICU	Large	Mar	0.117	0.114	0.120	0.117	0.112	0.121
Hospital	ICU	Large	Apr	0.062	0.056	0.067	0.062	0.059	0.068
Hospital	ICU	Large	May	0.035	0.026	0.045	0.032	0.023	0.038
Hospital	ICU	Large	Jun	0.030	0.017	0.050	0.029	0.019	0.048
Hospital	ICU	Medium	Feb	0.146	0.120	0.160	0.144	0.121	0.164
Hospital	ICU	Medium	Mar	0.100	0.091	0.108	0.100	0.092	0.109
Hospital	ICU	Medium	Apr	0.053	0.046	0.059	0.053	0.049	0.060

Hospital	ICU	Medium	May	0.030	0.021	0.040	0.028	0.019	0.037
Hospital	ICU	Medium	Jun	0.026	0.015	0.043	0.025	0.016	0.043
Hospital	ICU	Small	Feb	0.085	0.071	0.094	0.084	0.068	0.095
Hospital	ICU	Small	Mar	0.056	0.050	0.061	0.056	0.049	0.061
Hospital	ICU	Small	Apr	0.028	0.025	0.032	0.028	0.024	0.033
Hospital	ICU	Small	May	0.015	0.011	0.021	0.014	0.010	0.018
Hospital	ICU	Small	Jun	0.013	0.007	0.023	0.013	0.007	0.022
Hospital	Death	Large	Feb	0.264	0.243	0.287	0.263	0.245	0.293
Hospital	Death	Large	Mar	0.250	0.245	0.257	0.250	0.246	0.255
Hospital	Death	Large	Apr	0.201	0.194	0.209	0.201	0.192	0.209
Hospital	Death	Large	May	0.137	0.120	0.160	0.137	0.120	0.159
Hospital	Death	Large	Jun	0.067	0.041	0.090	0.066	0.045	0.097
Hospital	Death	Medium	Feb	0.292	0.267	0.317	0.291	0.269	0.322
Hospital	Death	Medium	Mar	0.275	0.266	0.290	0.275	0.263	0.286
Hospital	Death	Medium	Apr	0.221	0.209	0.234	0.220	0.207	0.231
Hospital	Death	Medium	May	0.151	0.131	0.172	0.151	0.131	0.175
Hospital	Death	Medium	Jun	0.074	0.045	0.098	0.073	0.054	0.106
Hospital	Death	Small	Feb	0.233	0.210	0.250	0.232	0.218	0.266
Hospital	Death	Small	Mar	0.212	0.203	0.220	0.212	0.202	0.222
Hospital	Death	Small	Apr	0.162	0.151	0.170	0.162	0.154	0.172
Hospital	Death	Small	May	0.107	0.094	0.125	0.107	0.092	0.125
Hospital	Death	Small	Jun	0.051	0.030	0.070	0.050	0.034	0.076
ICU	Post-ICU	Large	Feb	0.522	0.460	0.587	0.522	0.451	0.587
ICU	Post-ICU	Large	Mar	0.537	0.518	0.553	0.537	0.517	0.558
ICU	Post-ICU	Large	Apr	0.657	0.620	0.699	0.657	0.619	0.695
ICU	Post-ICU	Large	MayJun	0.730	0.604	0.827	0.728	0.633	0.818

ICU	Post-ICU	Medium	Feb	0.493	0.428	0.569	0.493	0.422	0.579
ICU	Post-ICU	Medium	Mar	0.508	0.463	0.541	0.508	0.468	0.558
ICU	Post-ICU	Medium	Apr	0.630	0.592	0.685	0.630	0.576	0.673
ICU	Post-ICU	Medium	MayJun	0.706	0.556	0.804	0.705	0.588	0.826
ICU	Post-ICU	Small	Feb	0.794	0.733	0.864	0.794	0.726	0.849
ICU	Post-ICU	Small	Mar	0.804	0.764	0.845	0.804	0.764	0.844
ICU	Post-ICU	Small	Apr	0.871	0.842	0.912	0.871	0.831	0.905
ICU	Post-ICU	Small	MayJun	0.905	0.835	0.945	0.905	0.841	0.950
ICU	Death	Large	Feb	0.478	0.413	0.540	0.478	0.413	0.549
ICU	Death	Large	Mar	0.463	0.447	0.482	0.463	0.442	0.483
ICU	Death	Large	Apr	0.343	0.301	0.380	0.343	0.305	0.381
ICU	Death	Large	MayJun	0.270	0.173	0.396	0.272	0.182	0.367
ICU	Death	Medium	Feb	0.507	0.431	0.572	0.507	0.421	0.578
ICU	Death	Medium	Mar	0.492	0.459	0.537	0.492	0.442	0.532
ICU	Death	Medium	Apr	0.370	0.315	0.408	0.370	0.327	0.424
ICU	Death	Medium	MayJun	0.294	0.196	0.444	0.295	0.174	0.412
ICU	Death	Small	Feb	0.206	0.136	0.267	0.206	0.151	0.274
ICU	Death	Small	Mar	0.196	0.155	0.236	0.196	0.156	0.236
ICU	Death	Small	Apr	0.129	0.088	0.158	0.129	0.095	0.169
ICU	Death	Small	MayJun	0.095	0.055	0.165	0.095	0.050	0.159
Post-ICU	Discharge	Large	Feb	0.685	0.597	0.750	0.687	0.581	0.766
Post-ICU	Discharge	Large	Mar	0.913	0.900	0.926	0.913	0.896	0.929
Post-ICU	Discharge	Large	Apr	0.909	0.841	0.938	0.909	0.880	0.935
Post-ICU	Discharge	Large	MayJun	0.854	0.732	0.939	0.851	0.718	0.938
Post-ICU	Discharge	Medium	Feb	0.673	0.506	0.769	0.674	0.514	0.785
Post-ICU	Discharge	Medium	Mar	0.908	0.863	0.934	0.908	0.872	0.935

Post-ICU	Discharge	Medium	Apr	0.904	0.814	0.944	0.904	0.843	0.933
Post-ICU	Discharge	Medium	MayJun	0.847	0.638	0.924	0.843	0.702	0.933
Post-ICU	Discharge	Small	Feb	0.763	0.631	0.884	0.763	0.614	0.868
Post-ICU	Discharge	Small	Mar	0.939	0.907	0.971	0.939	0.896	0.964
Post-ICU	Discharge	Small	Apr	0.937	0.884	0.967	0.936	0.877	0.966
Post-ICU	Discharge	Small	MayJun	0.897	0.757	0.963	0.894	0.776	0.953
Post-ICU	Death	Large	Feb	0.315	0.250	0.403	0.313	0.234	0.419
Post-ICU	Death	Large	Mar	0.087	0.074	0.100	0.087	0.071	0.104
Post-ICU	Death	Large	Apr	0.091	0.062	0.159	0.091	0.065	0.120
Post-ICU	Death	Large	MayJun	0.146	0.061	0.268	0.149	0.062	0.282
Post-ICU	Death	Medium	Feb	0.327	0.231	0.494	0.326	0.215	0.486
Post-ICU	Death	Medium	Mar	0.092	0.066	0.137	0.092	0.065	0.128
Post-ICU	Death	Medium	Apr	0.096	0.056	0.186	0.096	0.067	0.157
Post-ICU	Death	Medium	MayJun	0.153	0.076	0.362	0.157	0.067	0.298
Post-ICU	Death	Small	Feb	0.237	0.116	0.369	0.237	0.132	0.386
Post-ICU	Death	Small	Mar	0.061	0.029	0.093	0.061	0.036	0.104
Post-ICU	Death	Small	Apr	0.063	0.033	0.116	0.064	0.034	0.123
Post-ICU	Death	Small	MayJun	0.103	0.037	0.243	0.106	0.047	0.224
Hospital	Discharge	Large	Feb	0.567	0.551	0.584	0.570	0.537	0.590
Hospital	Discharge	Large	Mar	0.633	0.626	0.638	0.633	0.628	0.640
Hospital	Discharge	Large	Apr	0.737	0.728	0.746	0.737	0.728	0.745
Hospital	Discharge	Large	May	0.829	0.802	0.847	0.830	0.809	0.852
Hospital	Discharge	Large	Jun	0.903	0.874	0.930	0.905	0.872	0.923
Hospital	Discharge	Medium	Feb	0.563	0.542	0.586	0.565	0.537	0.588
Hospital	Discharge	Medium	Mar	0.625	0.606	0.638	0.625	0.612	0.638
Hospital	Discharge	Medium	Apr	0.726	0.709	0.741	0.727	0.711	0.742

Hospital	Discharge	Medium	May	0.819	0.792	0.839	0.821	0.796	0.842
Hospital	Discharge	Medium	Jun	0.900	0.871	0.929	0.902	0.869	0.922
Hospital	Discharge	Small	Feb	0.682	0.664	0.701	0.685	0.653	0.707
Hospital	Discharge	Small	Mar	0.731	0.723	0.742	0.732	0.721	0.740

Table A.24: Estimated probabilities (95% confidence intervals) of next events, given current state, by hospital bed capacity, month of admission and missing outcome assumption: (a) censoring at 1 day after last observed event; (b) ignoring missing outcomes.

Estimated probabilities of final events are given in Table A.25:

Final Event	Hospital Bed Capacity	Month	CENSORING			MISSING		
			Pr(Final Event)	Lower	Upper	Pr(Final Event)	Lower	Upper
Death	Large	Feb	0.372	0.346	0.394	0.370	0.347	0.389
Death	Large	Mar	0.310	0.304	0.316	0.310	0.304	0.316
Death	Large	Apr	0.226	0.219	0.235	0.226	0.215	0.234
Death	Large	May	0.150	0.132	0.172	0.150	0.132	0.167
Death	Large	Jun	0.078	0.059	0.099	0.077	0.060	0.100
Death	Medium	Feb	0.389	0.362	0.418	0.387	0.363	0.412
Death	Medium	Mar	0.329	0.317	0.342	0.329	0.322	0.343
Death	Medium	Apr	0.243	0.233	0.256	0.243	0.230	0.258
Death	Medium	May	0.163	0.146	0.188	0.163	0.145	0.182
Death	Medium	Jun	0.085	0.061	0.106	0.083	0.063	0.107
Death	Small	Feb	0.267	0.241	0.295	0.265	0.240	0.286
Death	Small	Mar	0.226	0.218	0.236	0.226	0.218	0.233
Death	Small	Apr	0.167	0.158	0.177	0.167	0.155	0.175
Death	Small	May	0.110	0.097	0.126	0.109	0.097	0.125
Death	Small	Jun	0.053	0.039	0.068	0.052	0.039	0.070

Discharge	Large	Feb	0.628	0.606	0.654	0.630	0.611	0.653
Discharge	Large	Mar	0.690	0.684	0.696	0.690	0.684	0.696
Discharge	Large	Apr	0.774	0.765	0.781	0.774	0.766	0.785
Discharge	Large	May	0.850	0.828	0.868	0.850	0.833	0.868
Discharge	Large	Jun	0.922	0.901	0.941	0.923	0.900	0.940
Discharge	Medium	Feb	0.611	0.582	0.638	0.613	0.588	0.637
Discharge	Medium	Mar	0.671	0.658	0.683	0.671	0.657	0.678
Discharge	Medium	Apr	0.757	0.744	0.767	0.757	0.742	0.770
Discharge	Medium	May	0.837	0.812	0.854	0.837	0.818	0.855
Discharge	Medium	Jun	0.915	0.894	0.939	0.917	0.893	0.937
Discharge	Small	Feb	0.733	0.705	0.759	0.735	0.714	0.760
Discharge	Small	Mar	0.774	0.764	0.782	0.774	0.767	0.782
Discharge	Small	Apr	0.833	0.823	0.842	0.833	0.825	0.845
Discharge	Small	May	0.890	0.874	0.903	0.891	0.875	0.903
Discharge	Small	Jun	0.947	0.932	0.961	0.948	0.930	0.961

Table A.25: Estimated probabilities (95% confidence intervals) of final events, given current state (hospital-fatality risks and complement), by hospital bed capacity, month of admission and missing outcome assumption: (a) censoring at 1 day after last observed event; (b) ignoring missing outcomes.

Estimated times to next events, under the censoring assumption only, are given in Table A.26:

From	Next Event	Hospital Bed Capacity	Month	Mean	95% CI of Mean		Median	95% CI of Median		25%-ile	95% CI of 25%-ile		75%-ile	95% CI of 75%-ile	
Hospital	Discharge	Large	Feb	28.3	26.1	30.6	17.9	16.6	19.1	6.4	5.9	6.8	39.2	36.5	41.7
	Discharge	Large	Mar	15.4	15.2	15.7	9.7	9.5	9.9	3.5	3.4	3.6	21.4	21.0	21.7
	Discharge	Large	Apr	15.2	14.8	15.8	9.6	9.3	9.8	3.4	3.3	3.5	21.0	20.5	21.6

	Discharge	Large	May	11.0	10.5	11.7	6.9	6.5	7.2	2.5	2.3	2.6	15.1	14.3	15.8
	Discharge	Large	Jun	7.7	6.8	8.6	4.8	4.5	5.3	1.7	1.6	1.9	10.6	9.9	11.7
	Discharge	Medium	Feb	32.5	29.5	35.0	20.5	18.9	22.2	7.4	6.8	8.0	45.0	41.5	48.7
	Discharge	Medium	Mar	17.7	17.0	18.4	11.2	10.8	11.6	4.0	3.9	4.2	24.5	23.8	25.5
	Discharge	Medium	Apr	17.4	16.7	18.0	11.0	10.6	11.5	3.9	3.8	4.1	24.1	23.3	25.1
	Discharge	Medium	May	12.6	11.9	13.3	7.9	7.4	8.4	2.8	2.7	3.0	17.4	16.3	18.4
	Discharge	Medium	Jun	8.8	8.1	9.7	5.6	5.2	6.2	2.0	1.9	2.2	12.2	11.4	13.5
	Discharge	Small	Feb	42.7	39.9	46.5	26.9	24.8	28.8	9.7	8.9	10.4	59.0	54.4	63.1
	Discharge	Small	Mar	23.3	22.8	23.9	14.7	14.3	15.0	5.3	5.1	5.4	32.2	31.4	33.0
	Discharge	Small	Apr	22.9	22.2	23.7	14.4	14.0	15.1	5.2	5.0	5.4	31.6	30.7	33.0
	Discharge	Small	May	16.5	15.4	17.5	10.4	9.7	11.0	3.7	3.5	4.0	22.8	21.4	24.2
	Discharge	Small	Jun	11.6	10.6	12.6	7.3	6.8	8.0	2.6	2.4	2.9	16.0	14.7	17.7
	ICU	Large	Feb	5.2	4.3	5.8	3.2	2.7	3.6	1.2	1.0	1.3	7.1	6.1	8.0
	ICU	Large	Mar	5.5	5.3	5.8	3.5	3.3	3.6	1.2	1.1	1.3	7.6	7.4	7.9
	ICU	Large	Apr	5.4	5.0	5.9	3.4	3.1	3.7	1.2	1.1	1.3	7.4	6.7	8.2
	ICU	Large	May	4.0	2.9	5.7	2.5	1.8	3.2	0.9	0.6	1.2	5.6	4.1	7.0
	ICU	Large	Jun	2.4	1.1	6.3	1.5	0.7	3.5	0.5	0.3	1.3	3.3	1.7	7.8
	ICU	Medium	Feb	5.6	4.6	6.7	3.5	3.0	4.2	1.3	1.1	1.5	7.8	6.7	9.3
	ICU	Medium	Mar	6.0	5.5	6.6	3.8	3.4	4.2	1.3	1.2	1.5	8.3	7.6	9.2
	ICU	Medium	Apr	5.8	5.1	6.9	3.7	3.3	4.3	1.3	1.1	1.5	8.1	7.2	9.3
	ICU	Medium	May	4.4	3.0	6.5	2.7	2.0	3.7	1.0	0.7	1.3	6.0	4.4	8.0
	ICU	Medium	Jun	2.6	1.2	5.6	1.6	0.8	3.6	0.6	0.3	1.3	3.6	1.8	7.9
	ICU	Small	Feb	6.2	5.3	7.4	3.9	3.2	4.4	1.4	1.1	1.6	8.6	7.2	9.8
	ICU	Small	Mar	6.6	6.1	7.4	4.1	3.7	4.7	1.5	1.3	1.7	9.1	8.3	10.3
	ICU	Small	Apr	6.4	5.5	7.1	4.0	3.4	4.7	1.4	1.2	1.7	8.9	7.6	10.3
	ICU	Small	May	4.8	3.8	6.4	3.0	2.1	3.8	1.1	0.7	1.3	6.7	4.7	8.3

	ICU	Small	Jun	2.8	1.0	6.3	1.8	0.9	4.3	0.6	0.3	1.5	3.9	2.0	9.5
	Death	Large	Feb	23.2	21.5	26.0	17.1	15.4	18.5	7.8	7.0	8.5	32.1	28.9	34.7
	Death	Large	Mar	8.1	7.9	8.3	6.0	5.8	6.1	2.7	2.6	2.8	11.2	10.9	11.5
	Death	Large	Apr	10.0	9.6	10.5	7.4	7.0	7.7	3.4	3.2	3.5	13.9	13.3	14.4
	Death	Large	May	9.7	8.6	10.7	7.2	6.3	7.7	3.3	2.9	3.6	13.5	11.9	14.6
	Death	Large	Jun	7.2	4.8	9.7	5.3	3.9	7.0	2.4	1.8	3.2	9.9	7.3	13.2
	Death	Medium	Feb	24.2	22.4	26.8	17.8	15.8	19.5	8.1	7.2	8.9	33.5	29.8	36.6
	Death	Medium	Mar	8.4	8.1	8.9	6.2	6.0	6.5	2.8	2.7	2.9	11.7	11.2	12.2
	Death	Medium	Apr	10.5	9.8	11.2	7.7	7.3	8.2	3.5	3.3	3.7	14.5	13.7	15.4
	Death	Medium	May	10.2	9.1	11.4	7.5	6.6	8.1	3.4	3.0	3.7	14.0	12.6	15.3
	Death	Medium	Jun	7.5	5.4	10.3	5.5	4.1	7.5	2.5	1.9	3.4	10.4	7.8	14.1
	Death	Small	Feb	28.0	25.7	31.0	20.6	18.6	22.4	9.4	8.4	10.2	38.7	35.0	42.1
	Death	Small	Mar	9.8	9.1	10.2	7.2	6.8	7.5	3.3	3.1	3.4	13.5	12.9	14.0
	Death	Small	Apr	12.1	11.5	12.7	8.9	8.4	9.3	4.1	3.8	4.3	16.7	15.9	17.6
	Death	Small	May	11.8	10.2	13.6	8.6	7.8	9.4	3.9	3.5	4.3	16.3	14.6	17.7
	Death	Small	Jun	8.7	6.1	11.5	6.4	4.7	8.5	2.9	2.2	3.9	12.0	8.8	16.0
ICU	Post-ICU	Large	Feb	14.6	12.0	16.8	11.1	9.7	12.8	5.3	4.6	6.2	20.2	17.7	23.6
	Post-ICU	Large	Mar	17.7	16.8	18.4	13.4	12.7	14.1	6.4	6.0	6.8	24.4	23.4	25.6
	Post-ICU	Large	Apr	14.8	13.9	16.4	11.2	10.2	12.2	5.3	4.8	5.9	20.3	18.4	22.1
	Post-ICU	Large	MayJun	10.5	7.8	14.0	7.9	5.7	10.5	3.8	2.7	5.0	14.5	10.5	19.1
	Post-ICU	Medium	Feb	14.6	11.6	16.6	11.1	9.1	12.6	5.3	4.4	6.1	20.1	16.5	22.8
	Post-ICU	Medium	Mar	17.7	16.5	19.9	13.4	12.2	14.8	6.4	5.7	7.0	24.4	22.3	27.1
	Post-ICU	Medium	Apr	14.7	13.2	16.2	11.1	9.9	12.9	5.3	4.7	6.0	20.3	17.8	23.8
	Post-ICU	Medium	MayJun	10.5	8.4	13.6	7.9	5.5	10.2	3.8	2.6	4.8	14.4	10.1	18.8
	Post-ICU	Small	Feb	13.3	11.2	15.6	10.1	8.8	11.7	4.8	4.2	5.6	18.3	15.9	21.4
	Post-ICU	Small	Mar	16.1	14.5	18.0	12.2	11.0	13.2	5.8	5.2	6.3	22.2	20.2	24.3

	Post-ICU	Small	Apr	13.4	11.9	14.9	10.1	9.2	11.4	4.8	4.3	5.5	18.5	16.6	20.9
	Post-ICU	Small	MayJun	9.5	7.2	11.8	7.2	5.0	9.2	3.4	2.4	4.4	13.1	9.2	16.9
	Death	Large	Feb	13.6	11.8	16.0	10.8	9.4	12.2	5.5	4.8	6.5	18.6	16.3	20.8
	Death	Large	Mar	12.2	11.6	12.6	9.6	9.0	10.0	4.9	4.6	5.2	16.7	15.8	17.4
	Death	Large	Apr	14.3	12.9	16.0	11.3	9.8	13.2	5.8	5.1	6.8	19.6	17.0	22.7
	Death	Large	MayJun	4.7	3.0	6.3	3.7	2.4	5.2	1.9	1.3	2.7	6.4	4.2	9.0
	Death	Medium	Feb	12.0	10.3	14.1	9.5	7.9	11.9	4.9	4.0	6.3	16.5	13.7	20.4
	Death	Medium	Mar	10.8	9.7	11.7	8.5	7.7	9.5	4.4	3.9	4.9	14.7	13.6	16.6
	Death	Medium	Apr	12.7	11.6	14.7	10.0	8.4	11.8	5.1	4.3	6.1	17.3	14.5	20.5
	Death	Medium	MayJun	4.1	2.8	6.0	3.3	2.0	4.5	1.7	1.1	2.4	5.6	3.5	7.9
	Death	Small	Feb	14.2	11.3	17.8	11.2	8.7	14.2	5.8	4.4	7.3	19.4	15.1	24.8
	Death	Small	Mar	12.7	10.5	15.1	10.0	7.8	12.5	5.1	4.1	6.4	17.3	13.5	21.6
	Death	Small	Apr	14.9	12.1	18.9	11.8	9.3	15.2	6.0	4.9	7.8	20.4	15.9	26.6
	Death	Small	MayJun	4.8	3.1	7.6	3.8	2.5	6.1	2.0	1.3	3.1	6.6	4.4	10.5
Post-ICU	Discharge	Large	Feb	22.6	19.5	26.9	19.2	16.7	22.6	11.3	9.7	13.3	30.3	26.3	35.7
	Discharge	Large	Mar	21.7	21.0	22.6	18.5	17.7	19.4	10.9	10.3	11.5	29.1	27.7	30.4
	Discharge	Large	Apr	18.8	17.0	20.2	16.0	15.1	17.4	9.4	8.8	10.2	25.2	23.8	27.4
	Discharge	Large	MayJun	12.8	10.2	15.3	11.0	8.9	13.6	6.4	5.2	8.1	17.2	14.1	21.3
	Discharge	Medium	Feb	26.8	24.1	30.9	22.8	19.4	27.3	13.4	11.3	16.1	35.9	30.7	42.5
	Discharge	Medium	Mar	25.8	23.8	27.9	22.0	19.6	23.6	12.9	11.5	14.0	34.5	30.8	36.8
	Discharge	Medium	Apr	22.3	19.4	25.4	19.0	17.2	21.3	11.2	10.1	12.7	29.9	27.1	33.3
	Discharge	Medium	MayJun	15.2	11.9	19.1	13.0	10.6	17.5	7.6	6.1	10.2	20.5	16.8	27.7
	Discharge	Small	Feb	27.8	23.5	32.7	23.7	19.9	28.6	13.9	11.6	16.8	37.3	31.8	44.4
	Discharge	Small	Mar	26.7	24.4	28.8	22.8	21.3	24.6	13.4	12.4	14.4	35.9	33.3	38.9
	Discharge	Small	Apr	23.1	21.4	26.3	19.7	17.6	21.9	11.6	10.4	12.9	31.0	27.6	34.6
	Discharge	Small	MayJun	15.8	11.9	22.7	13.5	11.0	16.9	7.9	6.5	9.8	21.2	17.3	26.7

	Death	Large	Feb	11.4	8.7	17.0	7.7	6.0	10.0	4.4	3.4	6.0	13.6	10.9	18.4
	Death	Large	Mar	13.3	11.2	17.0	8.9	7.6	10.5	5.1	4.3	6.3	15.8	13.6	19.1
	Death	Large	Apr	14.1	10.5	21.3	9.4	7.3	13.4	5.4	4.2	7.7	16.7	12.4	23.8
	Death	Large	MayJun	10.8	3.8	20.8	7.2	4.0	14.3	4.2	2.2	8.4	12.8	6.9	25.1
	Death	Medium	Feb	11.8	7.1	17.2	7.9	5.3	11.8	4.6	3.1	6.7	14.1	9.6	20.6
	Death	Medium	Mar	13.7	9.9	21.5	9.2	6.3	11.3	5.3	3.7	6.7	16.3	10.8	21.7
	Death	Medium	Apr	14.6	9.1	23.4	9.8	5.7	16.5	5.6	3.4	9.6	17.3	9.6	29.8
	Death	Medium	MayJun	11.2	6.8	23.3	7.5	3.7	14.1	4.3	2.0	7.8	13.3	6.5	26.5
	Death	Small	Feb	10.0	6.7	19.6	6.7	4.7	10.3	3.9	2.6	5.9	11.9	8.3	18.0
	Death	Small	Mar	11.6	8.4	20.5	7.7	5.3	11.3	4.5	3.0	6.5	13.8	9.6	21.0
	Death	Small	Apr	12.3	8.1	18.3	8.2	5.0	12.0	4.7	2.9	7.0	14.6	9.0	21.7
	Death	Small	MayJun	9.4	4.8	24.1	6.3	2.6	17.5	3.6	1.5	10.3	11.2	4.6	30.3

Table A.26: Summaries of times from current state to next event, conditional on experiencing that next event, by month of admission, assuming missing outcomes are censoring at 1 day after last observed event.

Estimated times to final events (total length of stay in hospital), by pathway through hospital, are given in Table A.27, under the censoring assumption only:

**Outcome**	**Pathway**	**Hospital Bed Capacity**	**Month**	**Mean**	**95% CI of Mean**		**Median**	**95% CI of Median**		**25%-ile**	**95% CI of 25%-ile**		**75%-ile**	**95% CI of 75%-ile**	
Death	Hospital-Death	Large	Feb	23.2	21.4	26.3	17.0	15.5	18.5	7.9	7.0	8.5	31.6	29.5	34.9
Death	Hospital-Death	Large	Mar	8.1	7.9	8.2	5.9	5.7	6.1	2.6	2.6	2.8	11.2	10.7	11.6
Death	Hospital-Death	Large	Apr	10.0	9.7	10.4	7.2	7.0	7.7	3.3	3.2	3.6	13.6	13.3	14.5
Death	Hospital-Death	Large	May	9.7	9.1	10.6	7.2	6.3	8.2	3.3	2.9	3.7	13.4	11.9	15.5
Death	Hospital-Death	Large	Jun	7.2	5.2	9.5	5.2	3.7	7.1	2.4	1.7	3.2	9.9	7.0	13.3
Death	Hospital-Death	Medium	Feb	24.2	21.6	27.8	17.9	16.4	19.4	8.2	7.5	9.0	33.4	30.9	36.7

Death	Hospital-Death	Medium	Mar	8.4	8.0	8.8	6.2	5.8	6.5	2.9	2.6	3.0	11.7	11.0	12.3
Death	Hospital-Death	Medium	Apr	10.5	9.7	11.0	7.7	7.3	8.3	3.5	3.3	3.8	14.5	13.8	15.6
Death	Hospital-Death	Medium	May	10.2	9.1	11.3	7.6	6.6	8.6	3.5	3.0	4.0	14.2	12.3	16.2
Death	Hospital-Death	Medium	Jun	7.5	5.4	9.8	5.5	4.0	7.8	2.5	1.8	3.5	10.3	7.4	14.6
Death	Hospital-Death	Small	Feb	28.0	25.6	31.4	20.3	19.1	21.9	9.2	8.7	10.2	38.7	35.7	41.9
Death	Hospital-Death	Small	Mar	9.8	9.3	10.2	7.3	6.8	7.5	3.3	3.1	3.5	13.6	12.8	14.2
Death	Hospital-Death	Small	Apr	12.1	11.3	12.9	8.8	8.3	9.5	4.1	3.8	4.4	16.5	15.7	17.8
Death	Hospital-Death	Small	May	11.8	10.8	13.1	8.6	7.5	9.9	3.9	3.4	4.5	15.9	14.2	18.5
Death	Hospital-Death	Small	Jun	8.7	6.1	11.4	6.3	4.6	8.7	2.9	2.1	4.1	11.9	8.5	16.5
Death	Hospital-ICU-Death	Large	Feb	18.8	16.9	20.5	16.0	14.3	17.7	9.7	8.5	10.6	25.1	22.4	27.9
Death	Hospital-ICU-Death	Large	Mar	17.7	17.1	18.3	15.2	14.9	15.6	9.1	8.8	9.5	23.7	23.0	24.5
Death	Hospital-ICU-Death	Large	Apr	19.7	17.7	21.0	16.6	15.5	18.1	9.8	9.2	10.8	26.1	24.0	28.0
Death	Hospital-ICU-Death	Large	May	8.7	6.6	12.2	7.4	6.0	9.7	4.4	3.6	5.8	11.6	9.6	15.1
Death	Hospital-ICU-Death	Large	Jun	7.0	4.9	14.3	6.0	3.8	10.3	3.6	2.3	5.9	9.3	6.0	16.8
Death	Hospital-ICU-Death	Medium	Feb	17.7	15.3	19.6	15.3	13.0	17.3	9.1	7.7	10.4	23.4	20.4	26.9
Death	Hospital-ICU-Death	Medium	Mar	16.8	15.9	17.8	14.4	13.3	15.4	8.7	7.9	9.3	22.3	20.7	24.0
Death	Hospital-ICU-Death	Medium	Apr	18.5	16.9	20.1	16.0	14.5	16.9	9.5	8.6	10.3	24.6	22.4	26.4
Death	Hospital-ICU-Death	Medium	May	8.5	6.3	12.0	7.1	5.6	9.3	4.2	3.2	5.5	11.4	9.2	14.7
Death	Hospital-ICU-Death	Medium	Jun	6.7	4.5	15.1	5.8	3.6	10.1	3.5	2.2	5.8	9.1	5.6	16.8
Death	Hospital-ICU-Death	Small	Feb	20.4	17.1	23.7	17.7	14.8	20.8	10.6	8.8	12.5	27.3	22.8	32.6
Death	Hospital-ICU-Death	Small	Mar	19.3	16.7	22.2	16.3	14.5	18.6	9.9	8.7	11.2	25.3	22.6	28.6
Death	Hospital-ICU-Death	Small	Apr	21.3	18.5	25.3	18.3	15.5	20.4	10.8	9.2	12.0	28.1	24.2	32.0
Death	Hospital-ICU-Death	Small	May	9.7	7.0	14.2	8.1	6.4	10.9	4.9	3.8	6.5	12.9	10.0	17.2
Death	Hospital-ICU-Death	Small	Jun	7.7	5.4	17.5	6.6	4.1	11.7	4.0	2.4	7.1	10.2	6.5	19.7
Death	Hospital-ICU-Post-ICU-Death	Large	Feb	31.2	26.4	35.3	27.3	24.9	30.8	18.3	16.4	20.8	39.4	36.8	45.1
Death	Hospital-ICU-Post-ICU-Death	Large	Mar	36.5	34.0	39.6	31.9	30.1	33.6	21.2	19.7	22.0	46.4	43.9	49.6

Death	Hospital-ICU-Post-ICU-Death	Large	Apr	34.2	29.9	40.6	29.3	24.8	35.4	19.9	16.6	24.0	43.4	35.8	53.1
Death	Hospital-ICU-Post-ICU-Death	Large	May	25.3	20.0	38.4	21.7	16.2	28.4	14.7	10.7	19.4	31.6	23.7	41.1
Death	Hospital-ICU-Post-ICU-Death	Large	Jun	23.6	19.1	35.3	20.0	15.4	27.4	13.4	9.9	18.1	29.7	22.7	40.2
Death	Hospital-ICU-Post-ICU-Death	Medium	Feb	32.1	26.6	38.4	28.1	24.0	34.3	18.7	15.9	23.1	40.6	34.7	49.6
Death	Hospital-ICU-Post-ICU-Death	Medium	Mar	37.4	32.8	45.7	32.3	28.9	37.6	21.6	19.2	25.4	47.2	42.2	54.4
Death	Hospital-ICU-Post-ICU-Death	Medium	Apr	35.1	29.9	44.8	30.6	25.5	36.3	20.3	17.2	24.4	44.2	37.1	53.2
Death	Hospital-ICU-Post-ICU-Death	Medium	May	26.0	21.3	40.2	22.5	16.4	28.3	15.0	10.9	19.4	32.8	24.1	41.5
Death	Hospital-ICU-Post-ICU-Death	Medium	Jun	24.2	20.6	38.5	20.6	15.7	27.6	13.7	10.2	18.4	30.3	23.3	40.5
Death	Hospital-ICU-Post-ICU-Death	Small	Feb	29.5	25.7	35.3	25.7	21.8	31.8	17.0	14.4	21.4	37.5	31.9	45.8
Death	Hospital-ICU-Post-ICU-Death	Small	Mar	34.2	30.2	41.0	30.0	25.7	35.3	20.1	16.8	23.7	44.2	38.0	51.8
Death	Hospital-ICU-Post-ICU-Death	Small	Apr	32.1	27.2	41.0	28.1	23.0	34.3	18.8	15.3	23.4	40.3	33.3	50.0
Death	Hospital-ICU-Post-ICU-Death	Small	May	23.8	19.3	36.6	20.9	16.3	30.4	14.1	10.6	20.7	30.1	23.9	44.8
Death	Hospital-ICU-Post-ICU-Death	Small	Jun	21.8	17.1	34.7	18.7	13.8	29.1	12.4	8.9	19.3	27.5	20.5	43.2
Discharge	Hospital-Discharge	Large	Feb	28.3	27.0	30.4	17.9	16.7	19.0	6.6	5.8	6.8	39.2	36.3	41.4
Discharge	Hospital-Discharge	Large	Mar	15.4	15.2	15.6	9.6	9.4	10.0	3.4	3.3	3.7	21.2	20.8	22.0
Discharge	Hospital-Discharge	Large	Apr	15.2	14.8	15.5	9.8	9.2	9.8	3.4	3.2	3.6	21.4	20.3	21.6
Discharge	Hospital-Discharge	Large	May	11.0	10.4	11.7	6.9	6.5	7.4	2.5	2.3	2.7	15.4	14.2	16.3
Discharge	Hospital-Discharge	Large	Jun	7.7	6.9	8.4	4.9	4.2	5.5	1.7	1.5	2.0	10.5	9.2	11.9
Discharge	Hospital-Discharge	Medium	Feb	32.5	31.0	35.1	20.0	19.2	21.7	7.3	6.9	7.9	44.6	42.1	47.4
Discharge	Hospital-Discharge	Medium	Mar	17.7	17.2	18.3	11.0	10.5	11.7	3.9	3.7	4.3	24.1	23.4	25.6
Discharge	Hospital-Discharge	Medium	Apr	17.4	16.8	18.1	10.9	10.3	11.4	3.9	3.7	4.1	24.3	22.9	25.2
Discharge	Hospital-Discharge	Medium	May	12.6	11.9	13.3	7.9	7.4	8.7	3.0	2.6	3.1	17.4	16.4	19.2
Discharge	Hospital-Discharge	Medium	Jun	8.8	7.9	9.6	5.6	4.8	6.3	2.0	1.7	2.2	12.1	10.4	13.7
Discharge	Hospital-Discharge	Small	Feb	42.7	40.6	46.2	26.4	25.1	28.6	9.2	9.0	10.1	58.9	55.1	62.2
Discharge	Hospital-Discharge	Small	Mar	23.3	22.6	23.9	14.9	13.9	15.3	5.4	5.0	5.5	32.6	30.9	33.5
Discharge	Hospital-Discharge	Small	Apr	22.9	21.8	23.7	14.3	13.4	15.1	5.2	4.9	5.5	32.1	29.7	32.8

Discharge	Hospital-Discharge	Small	May	16.5	15.4	17.8	10.2	9.7	11.1	3.7	3.5	4.1	22.2	21.6	24.6
Discharge	Hospital-Discharge	Small	Jun	11.6	10.4	12.8	7.3	6.4	8.1	2.6	2.3	2.9	16.0	14.1	17.7
Discharge	Hospital-ICU-Post-ICU-Discharge	Large	Feb	42.4	38.6	46.1	39.0	36.0	44.5	27.2	25.1	31.1	54.2	49.7	61.1
Discharge	Hospital-ICU-Post-ICU-Discharge	Large	Mar	44.9	43.4	46.0	41.3	40.1	42.5	28.8	27.6	29.7	57.5	55.5	58.8
Discharge	Hospital-ICU-Post-ICU-Discharge	Large	Apr	38.9	36.8	40.8	35.6	33.7	37.6	24.6	23.3	26.2	49.1	46.6	51.9
Discharge	Hospital-ICU-Post-ICU-Discharge	Large	May	27.4	23.3	34.0	24.9	21.0	31.0	17.2	14.5	21.3	34.8	29.2	43.2
Discharge	Hospital-ICU-Post-ICU-Discharge	Large	Jun	25.7	21.7	32.9	23.4	19.3	29.7	16.1	13.2	20.6	32.9	27.2	41.1
Discharge	Hospital-ICU-Post-ICU-Discharge	Medium	Feb	47.0	42.3	52.6	43.4	38.4	49.5	30.4	26.8	34.5	60.1	52.8	68.6
Discharge	Hospital-ICU-Post-ICU-Discharge	Medium	Mar	49.4	46.5	52.4	45.1	43.2	47.3	31.6	29.9	33.1	62.9	59.8	65.4
Discharge	Hospital-ICU-Post-ICU-Discharge	Medium	Apr	42.8	40.8	45.5	39.2	36.5	41.5	27.3	25.5	29.1	54.4	50.6	57.6
Discharge	Hospital-ICU-Post-ICU-Discharge	Medium	May	30.1	25.3	37.8	27.9	23.1	33.0	19.4	16.2	23.0	38.4	32.3	45.4
Discharge	Hospital-ICU-Post-ICU-Discharge	Medium	Jun	28.3	23.2	36.4	25.7	21.4	31.7	17.8	14.8	22.3	36.1	29.8	43.7
Discharge	Hospital-ICU-Post-ICU-Discharge	Small	Feb	47.3	41.9	52.3	43.2	39.7	50.2	30.8	27.9	35.1	60.1	55.0	69.5
Discharge	Hospital-ICU-Post-ICU-Discharge	Small	Mar	49.4	46.7	53.0	45.3	43.0	49.0	31.9	30.0	34.1	62.9	59.5	67.5
Discharge	Hospital-ICU-Post-ICU-	Small	Apr	43.0	39.2	46.6	39.6	36.1	42.6	28.0	25.3	30.1	54.7	50.0	58.9

	Discharge														
Discharge	Hospital-ICU-Post-ICU-Discharge	Small	May	30.2	25.4	38.0	28.1	22.6	33.7	19.5	15.8	23.5	38.8	31.3	46.3
Discharge	Hospital-ICU-Post-ICU-Discharge	Small	Jun	28.2	23.8	36.9	25.7	21.6	31.5	17.8	14.6	21.8	35.9	29.7	43.5
Death	Averaged over pathways	Large	Feb	22.8	21.1	24.4	17.4	16.4	19.2	8.6	8.2	9.6	30.4	29.0	33.6
Death	Averaged over pathways	Large	Mar	10.3	10.1	10.5	7.3	7.1	7.6	3.5	3.1	3.5	13.6	13.4	14.4
Death	Averaged over pathways	Large	Apr	11.3	10.9	11.8	7.8	7.8	8.7	3.5	3.5	4.0	15.9	14.7	16.4
Death	Averaged over pathways	Large	May	10.1	9.2	11.1	7.2	6.7	8.4	3.3	3.0	3.9	13.7	12.6	15.8
Death	Averaged over pathways	Large	Jun	7.8	6.2	10.4	5.7	4.7	7.6	2.6	2.2	3.6	10.8	8.6	14.6
Death	Averaged over pathways	Medium	Feb	23.4	21.6	25.3	17.5	16.5	19.1	8.8	8.0	9.6	32.1	29.8	34.3
Death	Averaged over pathways	Medium	Mar	10.1	9.7	10.6	7.1	6.9	7.8	3.4	3.1	3.5	13.9	13.0	14.6
Death	Averaged over pathways	Medium	Apr	11.4	11.1	12.0	8.3	7.7	8.8	3.8	3.4	4.1	15.4	14.6	16.6
Death	Averaged over pathways	Medium	May	10.4	9.5	11.5	7.6	6.7	9.0	3.5	3.1	4.2	13.3	12.7	16.6
Death	Averaged over pathways	Medium	Jun	8.0	6.3	10.7	5.4	4.8	7.7	2.6	2.2	3.4	10.8	8.8	14.7
Death	Averaged over pathways	Small	Feb	27.6	25.5	29.6	20.5	18.6	22.6	10.0	8.8	11.1	36.4	33.6	41.5
Death	Averaged over pathways	Small	Mar	10.5	10.1	11.1	7.6	7.0	8.1	3.5	3.2	3.8	14.3	13.6	15.3
Death	Averaged over pathways	Small	Apr	12.5	11.8	13.4	8.8	8.4	9.7	4.1	3.7	4.6	17.2	15.7	18.1
Death	Averaged over pathways	Small	May	11.9	10.8	13.5	8.4	7.8	10.1	3.8	3.5	4.8	16.3	14.5	19.0
Death	Averaged over pathways	Small	Jun	8.9	6.9	12.3	7.4	5.1	8.6	3.2	2.3	4.0	14.0	9.5	16.3
Discharge	Averaged over pathways	Large	Feb	29.7	27.3	31.2	20.8	19.1	21.7	7.4	6.8	7.9	42.6	39.6	44.2
Discharge	Averaged over pathways	Large	Mar	17.9	17.6	18.2	11.3	10.7	11.8	3.9	3.8	4.1	25.7	24.4	26.4
Discharge	Averaged over pathways	Large	Apr	16.3	15.9	16.6	10.8	10.0	10.7	3.7	3.5	3.8	23.7	22.1	23.7
Discharge	Averaged over pathways	Large	May	11.4	10.6	11.9	7.3	6.8	7.6	2.5	2.4	2.8	16.1	15.1	16.7
Discharge	Averaged over pathways	Large	Jun	8.0	7.6	8.8	5.0	4.6	5.5	1.8	1.6	2.0	11.1	10.2	12.2
Discharge	Averaged over pathways	Medium	Feb	33.7	31.1	36.0	22.7	21.2	24.8	8.0	7.6	9.3	46.6	44.3	50.5

Discharge	Averaged over pathways	Medium	Mar	19.9	19.3	20.7	12.8	12.2	13.4	4.5	4.1	4.8	28.4	27.0	29.5
Discharge	Averaged over pathways	Medium	Apr	18.4	17.9	19.1	11.6	11.0	12.3	4.1	4.0	4.4	25.6	24.8	27.3
Discharge	Averaged over pathways	Medium	May	12.9	12.1	13.6	8.3	7.6	8.8	2.9	2.7	3.2	18.2	17.0	19.4
Discharge	Averaged over pathways	Medium	Jun	9.1	8.6	10.1	5.7	5.1	6.2	2.0	1.8	2.3	12.7	11.5	14.1
Discharge	Averaged over pathways	Small	Feb	43.0	40.2	45.4	28.7	26.6	31.1	10.6	9.8	11.7	60.0	56.0	63.0
Discharge	Averaged over pathways	Small	Mar	24.7	24.0	25.5	16.1	15.3	16.8	5.8	5.4	5.9	34.8	33.7	36.3
Discharge	Averaged over pathways	Small	Apr	23.4	22.9	24.3	15.7	14.4	15.8	5.6	5.1	5.7	34.0	31.4	34.4
Discharge	Averaged over pathways	Small	May	16.7	15.4	17.7	10.4	9.9	11.6	3.8	3.5	4.1	23.1	21.6	25.0
Discharge	Averaged over pathways	Small	Jun	11.8	11.0	13.0	7.3	6.8	8.3	2.5	2.4	3.0	16.1	14.8	18.3

Table A.27: Summaries of times from hospital admission to final events (total length of stay), by pathway through hospital, hospital bed capacity and month of admission, conditional on experiencing that final event and assuming missing outcomes are censoring at 1 day after last observed event.

Estimated total lengths of stay in hospital by month of admission, averaged over pathways and final outcomes, are given in Table A.28, under the censoring assumption only:

**Hospital Bed Capacity**	**Month**	**Mean**	**95% CI of Mean**		**Median**	**95% CI of Median**		**25%-ile**	**95% CI of 25%-ile**		**75%-ile**	**95% CI of 75%-ile**	
Large	Feb	27.1	25.6	28.3	18.9	18.1	20.1	7.8	7.5	8.4	37.4	35.3	39.0
Large	Mar	15.5	15.3	15.7	9.7	9.3	9.8	3.8	3.5	3.9	21.8	20.3	21.5
Large	Apr	15.2	14.9	15.6	9.7	9.4	10.1	3.7	3.5	3.8	20.9	20.3	21.6
Large	May	11.2	10.7	11.5	7.3	6.7	7.7	2.8	2.5	2.9	15.4	14.4	16.5
Large	Jun	8.0	7.5	8.9	5.0	4.5	5.5	1.9	1.6	2.0	10.8	9.9	12.1
Medium	Feb	29.7	27.5	31.4	20.2	18.9	21.3	8.8	7.9	9.1	40.9	37.8	42.5
Medium	Mar	16.7	16.2	17.1	10.2	9.7	10.6	3.9	3.8	4.2	22.4	21.5	23.4
Medium	Apr	16.7	16.3	17.3	10.6	10.1	11.3	4.1	3.9	4.3	22.6	21.9	23.9

Medium	May	12.5	11.9	13.2	8.0	7.7	8.8	3.1	2.9	3.3	17.2	16.3	18.5
Medium	Jun	9.0	8.3	10.1	5.8	5.1	6.2	2.2	1.9	2.3	12.5	11.2	13.5
Small	Feb	38.9	36.8	41.3	26.1	24.3	27.3	10.6	9.7	11.2	53.2	49.1	55.2
Small	Mar	21.5	21.0	21.9	13.1	12.6	13.5	5.1	4.7	5.1	28.9	28.2	30.5
Small	Apr	21.6	21.1	22.3	13.3	13.1	14.1	5.0	4.8	5.3	29.6	28.7	30.6
Small	May	16.2	15.3	16.8	10.5	9.7	11.0	3.8	3.5	4.1	22.3	20.9	23.8
Small	Jun	11.6	10.7	13.1	7.5	6.6	8.1	2.7	2.3	2.9	16.5	14.3	17.5

Table A.28: Summaries of lengths of stay in hospital (total time in hospital), by hospital bed capacity and month of admission, averaged over pathways and final outcomes, assuming missing outcomes are censoring at 1 day after last observed event.

A.4 Definitions

Ageing index: the ratio of the number of elderly people (65+) compared to the number of young people (0-14 years old).

Old age dependency index: the ratio of the number of elderly people (65+), compared to the number of people of working age (15-64 years old).

Hospital-fatality risk: a measure of the severity of infection, is defined as the probability of death among cases who required hospitalization for medical reasons .

References

1. Regione Lombardia “Open Data”; Demografia; ex 6 https://www.dati.lombardia.it/stories/s/jx4t-xhet; Accessed 20 December 2020

2. Regione Lombardia “Open Data”; Mortalità; https://www.dati.lombardia.it/stories/s/av99-przx; Accessed 20 December 2020

3. Italian Ministry of Health, Ministerial Circular N°1997, 22 January 2020+

4. Welfare Directorate of Regione Lombardia, Protocol G1.2020.0002677, 23 January 2019

5. Welfare Directorate of Regione Lombardia, Regional Decree N° XI/2906; 8 March 2020
